# Engineered *Lactobacillus casei* targets the IgT-pIgR axis to confer mucosal protection against *Aeromonas veronii* in snakehead (*Channa argus*)

**DOI:** 10.3389/fimmu.2026.1759765

**Published:** 2026-03-23

**Authors:** Kui-Peng Gao, Jian-Ran Dou, Bing-Bing Ma, Tian-Bao Ma, Na Li, Ai-Dong Qian, Xiao-Feng Shan, Wu-Wen Sun, Lei Zhang, Di Zhang, Chun-Feng Wang, Dong-Xing Zhang

**Affiliations:** 1 College of Animal Science and Technology, Jilin Agricultural University, Changchun, China; 2 Engineerng Research Center of Microecological Vaccines (Drugs) for Major Animal Diseases, Ministry of Education, Jilin Agricultural University, Changchun, China; 3 Ministry of Agriculture and Rural Affairs of Mudanjiang, Mudanjiang, China

**Keywords:** *Aeromonas veronii*, *Channa argus*, immunoglobulin, *Lactobacillus casei*, mucosal immunity

## Abstract

**Introduction:**

*Aeromonas veronii* remains a pervasive aquatic pathogen precipitating catastrophic economic depletion and threats to global food security. Conventional therapeutic modalities are constrained by inadequate stability, prohibitive costs, and biosafety risks.

**Methods:**

To address such challenges, an engineered *Lactobacillus casei* strain was developed to constitutively express the *A. veronii* outer membrane protein OmpAI through a tandem promoter system to ensure optimized antigen delivery.

**Results:**

Anal intubation with engineered *L. casei* in *Channa argus* stimulated compartmentalized mucosal immunity, evidenced by a six-fold elevation in hindgut IgT transcripts, significant infiltration of IgT^+^ B cells, and pIgR mediated transcytosis, synchronized with systemic IgM activation. The probiotic treament accelerated pathogen clearance, improved survival rates to 42.9% compared to 12.5% in control group, and reorganized the commensal microbiome through a specific enrichment of beneficial *Firmicutes*.

**Discussion:**

This study elucidated a novel engineered-probiotic mucosal vaccination strategy for teleosts, providing a noninvasive, mucosal targeted immunoprophylactic strategy to decrease antibiotic dependency in aquaculture.

## 1 Introduction

*Aeromonas veronii* is a significant aquaculture pathogen, infecting diverse fish species and crustaceans, and inducing ulcerative lesions, septicemia, and mass mortality that incur substantial economic losses (1–3). Notably, *A. veronii* also threatens food safety due to its psychrotolerant toxin production, contaminating raw seafood products and potentially contributing to foodborne disease outbreaks (2, 4). Consequently, developing safe and efficient therapeutic delivery systems is imperative. Conventional strategies relying on active pharmaceutical ingredients (APIs) face limitations: invasive injections (intravenous or subcutaneous) cause stress, while soluble protein therapeutics exhibit poor stability, low efficiency, high cost, and dose-limiting toxicity during prolonged administration (5). Furthermore, microbial-based recombinant production raises biosafety concerns (6). In contrast, lactic acid bacteria (LAB) represent ideal antigen delivery vectors (7). Their Generally Recognized as Safe (GRAS) status, validated through extensive food-use history combined with intrinsic adjuvant properties, mucoadhesion (8–10), genetic tractability, and probiotic functions, render them particularly well-suited (11–14). Critically, as Gram-positive organisms, LAB lack endotoxic lipopolysaccharide (LPS), eliminating anaphylaxis risks inherent to Gram-negative systems (6). Through genetic engineering, heterologous proteins can be expressed and secreted or surface-displayed using constitutive or inducible promoters (Nisin-Controlled Expression system) (7, 15–17). Most importantly, LAB enable non-invasive mucosal immunization (oral or intranasal) (7), thereby circumventing injection-associated complications.

Engineered lactic acid bacteria (LAB) present compelling advantages as mucosal vaccine vectors against aquatic pathogens (18, 19). Particularly, oral or intranasal administration recapitulates the natural infection route of *A. veronii* (20), effectively eliciting dual mucosal and systemic immunity, demonstrated by elevated secretory IgA (sIgA) titers and antigen-specific lymphocyte proliferation (21, 22). In teleosts, the intestine serves as a pivotal site for antigen sampling and immune activation (23), wherein gut-associated lymphoid tissue (GALT) coordinates antigen-presenting cell (APC)-mediated responses (24–26). Mechanistically, LABs operate as multifaceted immunomodulators: (i) LAB-derived microbe-associated molecular patterns (MAMPs) engage host pattern recognition receptors (PRRs), activating innate immune cascades (27–29). (ii) Subsequent APC stimulation bridges adaptive immunity and mucosal antibody production (30). (iii) Competitive mucosal colonization excludes pathogens via niche occupation and antimicrobial secretion, concurrently enhancing microbiota equilibrium (31). Notably, engineered LAB vaccines exhibit superior safety profiles relative to attenuated or DNA-based platforms (32), establishing these systems as sustainable antibiotic-free interventions for aquaculture biosecurity (33).

Mucosa-associated lymphoid tissue (MALT) encompasses the teleost mucosal immune system, partitioned into intestinal (GALT), skin (SALT), gill (GIALT), and nasal compartments (34). Mucosal immunity within teleost species is primarily mediated by the immunoglobulin T (IgT) isotype, which serves as a functional analog to mammalian IgA (35–38). Pathogen exposure triggers local IgT production by mucosal B cells, with transport across epithelial barriers into mucus facilitated by the polymeric immunoglobulin receptor (pIgR) (39). Notably, teleost pIgR enables transcytosis of polymeric IgT and IgM through a J-chain-independent mechanism, as teleost genomes lack a J-chain homolog (40–42). Structural attributes of the IgT-pIgR axis are well-defined in model species like *Oncorhynchus mykiss*, while precise anatomical sites for immune induction vary considerably across diverse taxa (40, 43, 44). The potential role of the *Channa argus* hindgut as a specialized functional hub for compartmentalized IgT responses, reminiscent of mammalian Peyer’s patches, remains uncharacterized (45, 46). Clarification of such site-specific functions is vital for engineering mucosal vaccines designed to target inductive sites effectively in teleost (47).

The identified knowledge gap was addressed by the hypothesis that the *Channa argus* hindgut functions as a primary inductive site for the IgT-pIgR axis, enabling engineered probiotics to confer mucosal protection. *Lactobacillus casei* ATCC393, a model probiotic candidate within aquatic biotechnology, was employed as a biosafe delivery vehicle for targeted antigens. An engineered *L. casei* strain constitutively expressing the *A. veronii* outer membrane protein OmpAI was generated through a high-potency tandem promoter system. The kinetics of IgT^+^ B cell recruitment and pIgR-mediated transcytosis within the snakehead hindgut were elucidated, and the protective efficacy of the mucosal vaccination strategy against *A. veronii* challenge was evaluated. The research characterizes the hindgut as a critical lymphoid hub in *C. argus* and establishes a foundational framework for next-generation mucosal vaccines in aquaculture.

## 2 Materials and methods

### 2.1 Bacterial strains, plasmids, and growth conditions

*Lactobacillus casei* ATCC393 (wild-type), utilized as the expression host for *Aeromonas veronii* OmpAI, was cultivated in deMan Rogosa Sharpe broth (MRS; Solarbio, China) at 37 °C for 24–36 h under anaerobic conditions (10% H_2_, 10% CO_2_, 80% N_2_) within a sealed anaerobic chamber (Whitley DG250, UK). *A. veronii* TH0426, isolated from farmed *Pelteobagrus fulvidraco* (48), was maintained in *Luria-Bertani* (LB) broth at 30 °C. *Escherichia coli* MC1061 competent cells, employed for plasmid propagation, were grown aerobically in LB broth with agitation (200 rpm) at 37 °C. The *E. coli-lactobacillus* shuttle vector pPG612.1, harboring a surface-anchoring mechanism, was engineered for target protein expression. Specifically, the *Bacillus subtilis*-derived *pgsA* gene, encoding a structural anchoring matrix, was positioned downstream of the target insertion site. An upstream ssUSP secretion signal sequence was incorporated to direct transmembrane translocation of recombinant proteins. Chloramphenicol (Cm) was reconstituted in absolute ethanol to yield a 20 mg/mL stock solution and utilized at a final concentration of 10 μg/mL for plasmid selection. All remaining bacterial strains and plasmids referenced in this study are cataloged in **Table 1**.

**Table 1:** Strains and plasmid vectors applied in this study.

Strain or plasmid	Characteristics	Source
Strains		
*E. coli* DH5α	Host bacteria	Our laboratory preserves
*E. coli* MC1061	Host bacteria	Our laboratory preserves
*A. veronii* TH0426	Virulent strain	(48)
*L. casei* ATCC393	Host bacteria	Our laboratory preserves
Lc/pPG1-5P_ldh_-OmpAI-T_ldh_	Cm^r^, P_ldh_, T_ldh,_ OmpAI	This work
Plasmid		
pUC57-EGFP	Amp^r^, 3.2 kb	Our laboratory preserves
pPG612	Cm^r^, 4.2 kb	Our laboratory preserves
pPG1-nP-EGFP-T_ldh_	Cm^r^, P_tef_/P_pgm_/P_ldh_/P_pm_/P_eno_/T_ldh_	This work
pPG1-5P_ldh_-OmpAI-T_ldh_	Cm^r^, 5P_ldh_, T_ldh_, OmpAI	This work

### 2.2 Fish and ethics statement

Juvenile snakehead fish (*Channa argus*, mean body weight 45.50 ± 4.20 g, length 12.41 ± 1.40 cm) were procured from a certified aquaculture facility in Jilin Province, China. The fish were acclimatized for 14 days in 200 L recirculating aquaculture systems maintained at 28.0 ± 1.0 °C under 12-h photoperiod conditions. Commercial feed was administered twice daily at 1% total biomass, while approximately 30% water volume was replaced daily. The water quality parameters were rigorously maintained at: pH 7.8 ± 0.5, dissolved oxygen 5.6 ± 0.45 mg/L, nitrate<0.02 mg/L, and total ammonia 0.12 ± 0.01 mg/L. Specific-pathogen-free (SPF) male BALB/c mice (8 weeks old) were obtained from the Jilin Provincial Laboratory Animal Center. Mice were utilized for the production of polyclonal antibodies against snakehead immunoglobulins (IgT, IgM) and pIgR. All experimental procedures were conducted in strict accordance with NIH Guide for Care and Use of Laboratory Animals (NIH Publication No. 8023) following protocol approval by the Institutional Animal Care Committee.

### 2.3 Development of engineered *L. casei*

Based on the complete genome sequence of *L. casei* ATCC393 (GenBank accession: NZ_AP012544), open reading frames (ORFs) and flanking sequences for the *ldh* (L-lactate dehydrogenase, WP_025012771.1), *greA* (transcription elongation factor, WP_025013240.1), *pgm* (phosphoglycerate mutase, WP_025013311.1), and *tkt* (transketolase, WP_025013842.1) genes were identified. Subsequently, promoter regions within these sequences were predicted in silico utilizing the BDGP Neural Network Promoter Prediction tool and the SoftBerry Promoter Prediction resource (http://www.softberry.com). Consequently, specific primers targeting the predicted promoter sequences were designed using Primer Premier 6.0 software, and oligonucleotide sequences are detailed in **Table 2**. All primers were synthesized by Sangon Biotech Co., Ltd. (Shanghai, China).

**Table 2:** The primers sequence for PCR amplification.

Primer	Sequence (5’-3’)	Restriction site
P_tef_-F	**CCCGGG**GATCAGGAAATTAAAATTG	*Sma* I
P_tef_-R	**GATATC**GTAAATTTCCTCCTTTAAA	*EcoR* V
P_pgm_-F	**CCCGGG**AATTCAGCAATTTAGCCTT	*Sma* I
P_pgm_-R	**ACTAGT**CATATGGTAAATTTCCTCCT	*Spe* I
P_ldh_-F	**CCCGGG**GATAAAATTTCTAATGATT	*Sma* I
P_ldh_-R	**ACTAGT**CATATGGTAAATTTCCTCCT	*Spe* I
P_pm_-F	**CCCGGG**GAAAAAGAAAATGTTTTTG	*Sma* I
P_pm_-R	**GATATC**TGTAACCGTCCTCCTCACTA	*EcoR* V
P_eno_-F	**CCCGGG**GCACTGATTCTTTTTTCAGT	*Sma* I
P_eno_-R	**GATATC**AATAATTAACCTCCTCTAAT	*EcoR* V
T_ldh_-F	TTTGGCTTATGAGAGAAGAT	
T_ldh_-R	**GATATC**GCAAAAAGGCCATCCGTCA	*Ec*o*R* V
EGFP/P-F	AGGAGGAAATTTACCATATGATGGTGAGCAAGGGCGAGGA	
EGFP/P-R	CTTGTACAGCTCGTCCATGCCGAGA	
IgT/CH2-4/F	CG**GAATTC**CCAAGTGTGTTTCCTCTGATACAAT	*EcoR* I
IgT/CH2-4/R	CC**AAGCTT** GACAATGACTTTAGTAGCATTAACC	*Hind* III
IgM/CH2-4/F	AA**GATATC**GGTTGCCAACTCTTAAAGTGTTTGC	*EcoR* V
IgM/CH2-4/R	CC**AAGCTT** TGACTTTAGTAGCATTAACCAAAGA	*Hind* III
pIgR/ID5-F	AA**GATATC**GTGGTGAAGAGCGAACTGATTGGGG	*EcoR* V
pIgR/ID5-R	CC**AAGCTT** CAGCACGTGCACGGCTTCCTGTTTT	*Hind* III
OmpAI-F	TTTTAAAGGAGGAAATTTACATGATGAAAATGGCTCCTTCCA	
OmpAI-R	TTACTTCTGAACTTCTTGTACGCCA	

The bases underlined represent the sequence of enzyme digestion sites.

Genomic DNA of *L. casei* ATCC393 was subsequently isolated using the EasyPure Genomic DNA Kit (Omega Bio-tek). Target promoter fragments (Ptef, Ppgm, Pldh, Ppm, Peno) and the Tldh terminator were amplified from the extracted genomic template via PCR. Expression cassettes, designated nP-EGFP-T_ldh_ (where n denotes the number of identical promoters), were assembled by fusing the enhanced green fluorescent protein (EGFP) reporter gene with varying promoter combinations and the Tldh terminator. These cassettes and the pPG-1 vector backbone were digested independently with *Sma* I and *Eco*R V at 37 °C for 3.5 h. Digested fragments were purified and ligated into the linearized pPG-1 plasmid using T4 DNA Ligase (TransGen Biotech) at 25 °C for 6 h. The resultant pPG1-nP-EGFP-T_ldh_ ligation products were transformed into competent *L. casei* ATCC393 cells by electroporation (1.8 kV, 200 Ω, 25 μF). Transformed cells were resuscitated in MRS broth for 2 h at 37 °C anaerobically, followed by plating onto MRS agar supplemented with 5 μg/mL chloramphenicol. Chloramphenicol-resistant colonies were selected after 48 h incubation anaerobically. Plasmids from transformants were isolated and sequence-verified (Sangon Biotech) to confirm cassette integrity and orientation. The OmpAI gene fragment, amplified from *A. veronii* genomic DNA, replaced the EGFP gene within the verified pPG1-5P_ldh_-EGFP-T_ldh_ vector. The recombinant plasmid pPG1-5P_ldh_-OmpAI-T_ldh_ was confirmed by restriction enzyme analysis, bidirectional Sanger sequencing, and transformation into *L. casei* ATCC393 as described above. For subsequent experiments, *L. casei* harboring the empty pPG612 plasmid (Lc/pPG612) served as the vector control, while untransformed *L. casei* ATCC393 was used as the negative control.

### 2.4 Preparation of polyclonal antibodies against IgT, IgM and pIgR

The constant heavy chain domains 2-4 (CH2-4) from IgT, IgM genes, and immunoglobulin domain 5 (ID5) from pIgR, were cloned and expressed in a prokaryotic system. Specifically, the target sequences were amplified through polymerase chain reaction (PCR) using specific primers. Amplified products were subsequently ligated into the pET-28a(+) vector harboring a hexahistidine tag. Recombinant plasmids were transformed into *Escherichia coli* BL21(DE3) competent cells, and protein expression was induced with 1 mM isopropyl β-D-1-thiogalactopyranoside (IPTG) at 37 °C for 4 h. Expressed proteins were purified via nickel-nitrilotriacetic acid (Ni-NTA) affinity chromatography employing the HisPur™ Ni-NTA Purification Kit (Thermo Scientific), adhering to the provided instructions, and concentrations were quantified using the bicinchoninic acid (BCA) protein assay kit. Purified recombinant proteins (IgT CH2-4, IgM CH2-4, and pIgR ID5) were prepared for immunization by emulsification of approximately 600 μg of each protein with complete Freund’s adjuvant for the initial dose and incomplete Freund’s adjuvant for subsequent doses. The immunization protocol consisted of a primary injection followed by four booster administrations at 14-day intervals. Additionally, serum antibody titers were assessed by enzyme-linked immunosorbent assay (ELISA) after each immunization to confirm immune response progression.

### 2.5 Western blot and co-immunoprecipitation

Bacterial pellets were harvested by centrifugation (5, 000×g, 10 min, 4 °C) and washed thrice with sterile phosphate-buffered saline (PBS, pH 7.4), and then treated with 100 mg/mL lysozyme at 37 °C for 1 h. After solubilization, samples were mixed with 6× Laemmli loading buffer, denatured at 95 °C for 10 min, and resolved electrophoretically on 10% SDS-polyacrylamide gels. Proteins were transferred onto polyvinylidene difluoride (PVDF) membranes using semi-dry transfer (25 V, 30 min).

Snakehead serum and intestinal mucus samples were separated on 4-15% gradient SDS-PAGE gels prior to PVDF transfer. All membranes were blocked with 5% (w/v) skim milk in PBS containing 0.1% Tween-20 (PBST) at 4 °C for 10 h. Membranes were subsequently incubated with anti-EGFP (1:2, 000, Bioss), anti-IgT (1:200, rabbit polyclonal) and anti-OmpAI (1:200, mouse polyclonal) antibodies diluted in PBST at ambient temperature for 2 h, followed by HRP-conjugated goat anti-rabbit or mouse IgG (1:2, 000 dilution in PBST) for 1 h. Protein bands were visualized using ECL Plus substrate (Thermo Scientific) according to manufacturer’s specifications. The band intensities were quantified using ImageJ software.

To elucidate interactions involving the secretory component (SC), co-immunoprecipitation was performed using anti-snakehead IgT and anti-pIgR antibodies. Briefly, 200 μL intestinal mucus aliquots were incubated with 100 μL of either mouse anti-IgT or rabbit anti-pIgR antibody at 4 °C overnight. Species-matched pre-immune sera served as negative controls. Protein G Agarose beads (20 μL slurry, Thermo Fisher) were added to each mixture and incubated at 4 °C for 1 h with gentle agitation. Beads were pelleted (1, 000×g, 2 min) and washed five times with ice-cold PBS. Ultimately, bound complexes were eluted using Laemmli sample buffer at 95 °C for 5 min. Primary antibodies employed were anti-IgT (1:200, rabbit polyclonal), anti-IgM (1:200, rabbit polyclonal), anti-IgM (1:200, mouse polyclonal), and anti-pIgR (1:2000, mouse polyclonal). Secondary antibodies used were HRP-conjugated goat anti-rabbit IgG (1:2000, Bioss) and HRP-conjugated goat anti-mouse IgG (1:2000, Bioss).

### 2.6 Immunofluorescence staining analysis

EGFP expression in wild-type *L. casei*, Lc/pPG1-EGFP, and Lc/pPG1-nP-EGFP-T_ldh_ strains was initially assessed by direct fluorescence microscopy. For quantitative analysis, bacterial cultures were grown for 12 h, subsequently pelleted by centrifugation (8, 000×g, 4 °C, 10 min), and washed twice with PBS. Cell pellets were resuspended in fresh PBS to a standardized optical density. Optical density at 600 nm (OD_600_) was measured using a UV-Vis spectrophotometer, while fluorescence intensity (excitation: 488 nm, emission: 517 nm) was quantified using a fluorescence spectrophotometer. Relative fluorescence units (RFU) were calculated using the formula: RFU=(Sample Fluorescence Intensity/Sample OD_600_)-(Wild-type Fluorescence Intensity/Wild-type OD_600_). Surface expression of OmpAI by Lc/pPG1-5P_ldh_-OmpAI-T_ldh_ was evaluated differently: PBS-washed cells were sequentially incubated with primary antibody (37 °C, 1 h) followed by fluorophore-conjugated secondary antibody (37 °C, 1 h), mounted on glass slides using anti-fade mounting medium, and ultimately examined by fluorescence microscopy.

Hindgut and head kidney tissues harvested from snakehead were cryosectioned at a thickness of 6 μm. Sections were fixed with 4% paraformaldehyde (PFA) for 5 min, air-dried, and permeabilized with 0.3% Triton X-100 in PBS at 37 °C for 3 h. Then, non-specific binding sites were blocked using 10% (v/v) normal goat serum in PBS for 4 h at room temperature. Subsequently, sections were incubated with primary antibodies diluted in blocking buffer at 4 °C overnight. After thoroughly washed and sections were incubated with appropriate fluorophore-conjugated secondary antibodies for 2 h at room temperature, protected from light. Finally, nuclei were counterstained with 4’, 6-diamidino-2-phenylindole (DAPI, 1 µg/mL) for 5 min in the dark, mounted with anti-fade reagent, and imaged using a confocal laser scanning microscope.

Intestinal leukocytes were washed in PBS, fixed with 4% PFA for 15 min at room temperature, and blocked with a solution containing 10% normal goat serum and 0.03% Triton X-100 in PBS for 2 h. The cells were stained with primary antibodies (overnight, 4 °C) followed by fluorophore-conjugated secondary antibodies (2 h, room temperature, dark). The primary antibodies used were anti-IgT (1:2000, rabbit polyclonal), anti-IgM (1:2000, mouse polyclonal), anti-pIgR (1:2000, mouse polyclonal), anti-OmpAI (1:200, mouse polyclonal). Secondary antibodies used were FITC-conjugated goat anti-rabbit IgG (1:100), PE-conjugated goat anti-mouse IgG (1:100), FITC-conjugated goat anti-mouse IgG (1:100).

### 2.7 RNA isolation and gene expression analysis by qRT-PCR

The cultured engineered probiotics were collected by centrifugation (8, 000×g, 10 min, 4 °C) during mid-logarithmic growth phase at 12 h, 18 h, and 24 h post-inoculation. Total RNA was extracted from harvested cells using the RNAPure Bacteria Kit (BioFlux) according to the manufacturer’s instructions, with RNA integrity verified through spectrophotometry (A260/A280 ratio≥1.8). Complementary DNA synthesis was performed using 1 μg of total RNA and the M-MuLV First Strand cDNA Synthesis Kit (Sangon Biotech) with random hexamer primers. Similarly, the collected posterior intestine, head kidney and spleen tissues were lysed for the extraction of total RNA. The expression levels of IgT, IgM, pIgR, BCRα, IL-6, IL-10, IL-1β and TLR3 genes in the tissues were detected.

Quantitative real-time PCR (qPCR) analysis was ultimately conducted using SYBR Green Master Mix on a QuantStudio 5 system (Applied Biosystems), with reaction conditions as follows: initial denaturation at 95 °C for 3 min, followed by 40 cycles of 95 °C for 15 s and 60 °C for 30 s. The expression of the target gene in the normalized recombinant strain was normalized using the Ct (ΔΔCt) method with the endogenous internal reference gene dnaA, and the β-actin gene was used as the internal reference gene of the tissue. All primer sequences are documented in **Table 3**.

**Table 3:** The primers sequence for qPCR.

Primer	Sequence (5’-3’)	GenBank
*dnaA*-F	TCTGTTTATTTATGGTGGCG	CP_039707.1
*dnaA*-R	CTGCGGTCATCAAGTTTCA	
EGFP-F	ATCATGGCCGACAAGCAGAA	MN_443913.1
EGFP-R	TCTCGTTGGGGTCTTTGCTC	
*β-actin*-F	TTGAGCAGGAGATGGGAACCG	XM_016491832.1
*β-actin*-R	AGAGCCTCAGGGCAACGGAAA	
IgT-F	CTTTATGCTGCGTCCAGTAGAAC	XM_041957132.1
IgT-R	GCCAAGACACATAAGCCTCCTG	
IL-6-F	ATGCCAAAGACTCTCACGCA	XM_067489093.1
IL-6-R	TCTGGATCAGGTCGCTCTCA	
pIgR-F	CTTTGCTGGTGTGTGCTTCG	XM_067529076.1
pIgR-R	CTTGTCTTAACGCAGTATTCTCCTTG	
BCR*α*-F	TCCCTCCGTCCAGTACTGAG	XM_034144300.1
BCR*α*-R	GCTGGTAGAGATCCCACTGC	
IL-10-F	CAGTGCAGAAGAGTCGACTGCAA	XM_042766262.1
IL-10-R	CGCTTGAGATCCTGAAATATA	
IL-1*β*-F	GTTTACCTGAACATGTCGGC	XM_044197347.1
IL-1*β*-R	AGGGTGCTGATGTTCAGCCC	
TLR3-F	CATGTGAGACAGCTGAGGCA	XM_067509996.1
TLR3-R	CCTGCAAGAACTCCTGCTGA	

### 2.8 Animal studies

Healthy snakeheads (150 ± 15 g) were randomly allocated into three experimental groups (n=50 fish/group): Blank control (sterile PBS), Wild-type *L. casei* ATCC393, and engineered probiotic Lc/pPG1-5P_ldh_-OmpAI-T_ldh_. To evaluate the immunogenicity of the vaccine candidate while minimizing variability associated with oral feed intake hierarchies and gastric hydrolysis, anal intubation was employed as a standardized delivery model for this mechanistic study. This method ensures the delivery of a precise, known bacterial load directly to the hindgut lymphoid tissues. Fish underwent primary immunization via daily anal intubation with 2×10^8^ CFU of bacterial suspensions in 100 µL sterile PBS for five consecutive days. An identical booster immunization regimen was administered during days 19-23.

Samples were collected at three critical time points: pre-immunization baseline (day 0), post-primary immunization (day 6), and post-booster immunization (day 28). At each interval, five randomly selected specimens per group (n=5) were immersed in MS-222 (250 mg/L) until opercular movements ceased, followed by thorough surface sterilization using 75% ethanol swabs. Serum, intestinal mucus, and head kidney tissues were aseptically harvested. Specifically, blood samples were obtained via caudal venipuncture, allowed to clot at 4 °C for 2 h, and centrifuged (3, 000×g, 15 min, 4 °C) to isolate serum. Intestinal mucus was collected by gentle luminal scraping with sterile spatulas, suspended in protease inhibitor cocktail, and clarified by centrifugation (12, 000×g, 10 min, 4 °C). Head kidney tissues were immediately snap-frozen in liquid nitrogen for subsequent analysis.

### 2.9 Flow cytometry

Intestinal leukocytes were isolated from gut-associated lymphoid tissue (GALT) according to an established protocol (39). Briefly, intestinal tissue fragments were incubated in Dulbecco’s Modified Eagle Medium (DMEM) supplemented with 5% (v/v) FBS, 100 U/mL penicillin, and 100 μg/mL streptomycin at 4 °C for 30 min under gentle agitation (150 rpm). Next, samples were washed in PBS containing 0.37 mg/mL EDTA and 0.14 mg/mL dithiothreitol (DTT) for 30 min. Thereafter, enzymatic digestion was performed using collagenase type IV (1 mg/mL) at 20 °C for 2 h with continuous orbital shaking. The cellular pellets were collected by centrifugation (300×g, 10 min), washed twice with serum-free DMEM, and resuspended in PBS. Then, discontinuous Percoll density gradients (63% and 40%) were employed for leukocyte enrichment. Centrifugation was conducted at 400×g for 30 min at 4 °C. Leukocytes localized at the 40%-63% interphase were harvested, washed extensively with DMEM containing 5% FBS, and resuspended in complete medium for subsequent analyses.

Isolated intestinal and head kidney leukocytes (1×10^6^ cells) were stained with rabbit anti-IgT, mouse anti-IgM, mouse anti-pIgR at 4 °C for 1 h. After three washes with PBS containing 5% FBS, FITC-conjugated goat anti-rabbit IgG (1:2000 dilution) and PE-conjugated goat anti-mouse IgG (1:2000 dilution) secondary antibodies were incubated for 45 min at 37 °C. The cells underwent three additional washes prior to analysis using a BD FACSCanto II flow cytometer. Data processing was performed with FlowJo software (Version 10.8.1).

### 2.10 Survival study

On day 40 post-immunization, fish from each group (n=30) were challenged through 30-min immersion in *A. veronii* TH0426 suspension (2×10^7^ CFU/mL in sterile PBS). The PBS-treated fish received identical immersion treatment as mock-challenged controls. All experimental groups were housed in 200-L recirculating aquaculture systems maintained at 28.0 ± 0.5 °C with stocking densities not exceeding 1.5 kg/m³. Continuous clinical monitoring was implemented with twice daily mortality observation. Following the 12-day observation period post-challenge, survival rates were quantified through Kaplan-Meier analysis. Necropsy and bacteriological examination were additionally performed on all mortalities to confirm *A. veronii* etiology.

### 2.11 Tissue culture

Perfused hindgut, head kidney, and spleen tissues (50 mg/sample) were surface-sterilized through immersion in 70% ethanol for 2 min. Next, three sequential washes were performed with sterile PBS (pH 7.4) for 5 min per wash. Tissue explants were aseptically transferred to 24-well plates containing DMEM supplemented with 10% (v/v) FBS, 100 U/mL penicillin, and 100 μg/mL streptomycin. Cultures were maintained at 28 °C in a humidified atmosphere with 5% CO_2_, with 500 μL medium per well replenished every 48 h. Following a 7-day incubation period, conditioned supernatants were harvested following centrifugation at 300×g for 10 min at 4 °C to remove cellular debris. Clarified supernatants were immediately aliquoted and stored at -80 °C until further analysis.

### 2.12 Pull-down assay

Recombinant His-tagged OmpAI (5 μg) was immobilized onto Ni-NTA agarose beads (Qiagen) through incubation in binding buffer (50 mM NaH_2_PO_4_, 300 mM NaCl, 10 mM imidazole, pH 8.0) at 4 °C for 30 min with gentle rotation. The affinity matrix was incubated with 100 μL of pre-cleared sample lysates (serum, mucosal secretions, or tissue culture supernatants) at 4 °C for 4 h with constant agitation. The matrix underwent five sequential washes with ice-cold wash buffer (50 mM NaH_2_PO_4_, 300 mM NaCl, 20 mM imidazole, pH 8.0) using 1 mL per wash and centrifugation at 500×g for 2 min. Then, non-specific binding sites were blocked through incubation with binding buffer containing 10% (w/v) bovine serum albumin (BSA) for 1 h at room temperature. Finally, bound immunoglobulins were eluted using 2× Laemmli sample buffer containing 5% β-mercaptoethanol at 95 °C for 5 min. Eluates were subjected to SDS-PAGE under reducing conditions, and immunoblotting using rabbit anti-IgT (1:200) and mouse anti-IgM (1:100) primary antibodies with species-appropriate HRP-conjugated secondary antibodies (1:2000).

### 2.13 Enzyme-linked immune sorbent assay

Recombinant OmpAI (20 μg/mL in 50 mM carbonate-bicarbonate buffer, pH 9.6) was immobilized onto 96-well microplates through overnight incubation at 4 °C (100 μL/well). The plates were blocked with 8% (w/v) skim milk powder in PBS (200 μL/well) for 2 h at 37 °C. After three washes with PBST, serum or skin mucus samples were diluted in PBST and added in technical triplicates (100 μL/well). Next, pre-immune sera from naïve specimens were included as negative controls. Thereafter, plates were incubated with rabbit anti-IgT or mouse anti-IgM (1:500 in PBST/1% BSA) for 2 h at 37 °C. After additional PBST washes, species-appropriate HRP-conjugated secondary antibodies (1:2000 in PBST/1% BSA) were applied (100 μL/well) for 1 h at 37 °C. Enzymatic reactions were developed using TMB substrate for 30 min at room temperature, stopped with 2 M H_2_SO_4_ (50 μL/well), and absorbance was measured at 405 nm using a SpectraMax microplate reader. Endpoint titers were defined as the highest sample dilution yielding optical density (OD) values≥2 standard deviations above the mean of negative control wells (n=8 per plate).

### 2.14 Microbiome (16S) sequencing and analysis

After Exclude low confidence (ELC) treatments, fecal samples (n=5 fish/group) were obtained from colon tissue, and preserved in liquid nitrogen for 16S gene sequencing and analysis. Briefly, genomic DNA was extracted from colonic fecal samples utilizing the Stool Genomic DNA Extraction Kit (Macklin, China), and the quality of the extracted DNA was assessed using 1% agarose gel electrophoresis. The assessment of the purity and concentration of the DNA samples was conducted utilizing the NanoDrop 2000 UV-Vis spectrophotometric system (Thermo Fisher Scientific). Targeted amplification of the V3-V4 hypervariable domains within prokaryotic 16S ribosomal RNA genes was conducted via thermal cycler-mediated PCR, with the degenerate primer set 338F (5’-ACTCCTACGGGAGGCAGCA-3’) and 806R (5’-GGACTACHVGGGTWTCTAAT-3’). Purified amplicons were isolated using a gel-based DNA extraction system (Macklin), normalized to equimolar ratios, and subjected to high-throughput sequencing via either Illumina NovaSeq/MiSeq or PacBio Sequel II systems. Sequences with similarity were categorized into operational taxonomic units (OTUs) through alignment at a threshold of 97% sequence identity. The raw sequences were archived in FASTQ format, and the data analysis was conducted utilizing the Personalbio Cloud Platform (https://www.genescloud.cn/home).

### 2.15 Statistical analysis

All statistical analyses were performed using Microsoft Excel and GraphPad Prism 9.0 software. The figure legends provide a description of the *P* values and the statistical analyses. Intergroup comparisons employed parametric ANOVA models (one-way or two-way) with Tukey’s analysis. Experimental data are expressed as mean ± standard error of the mean (SEM).

## 3 Results

### 3.1 Engineered tandem Pldh promoter platform drives high-level constitutive expression in *L. casei*

**Figure 1:**
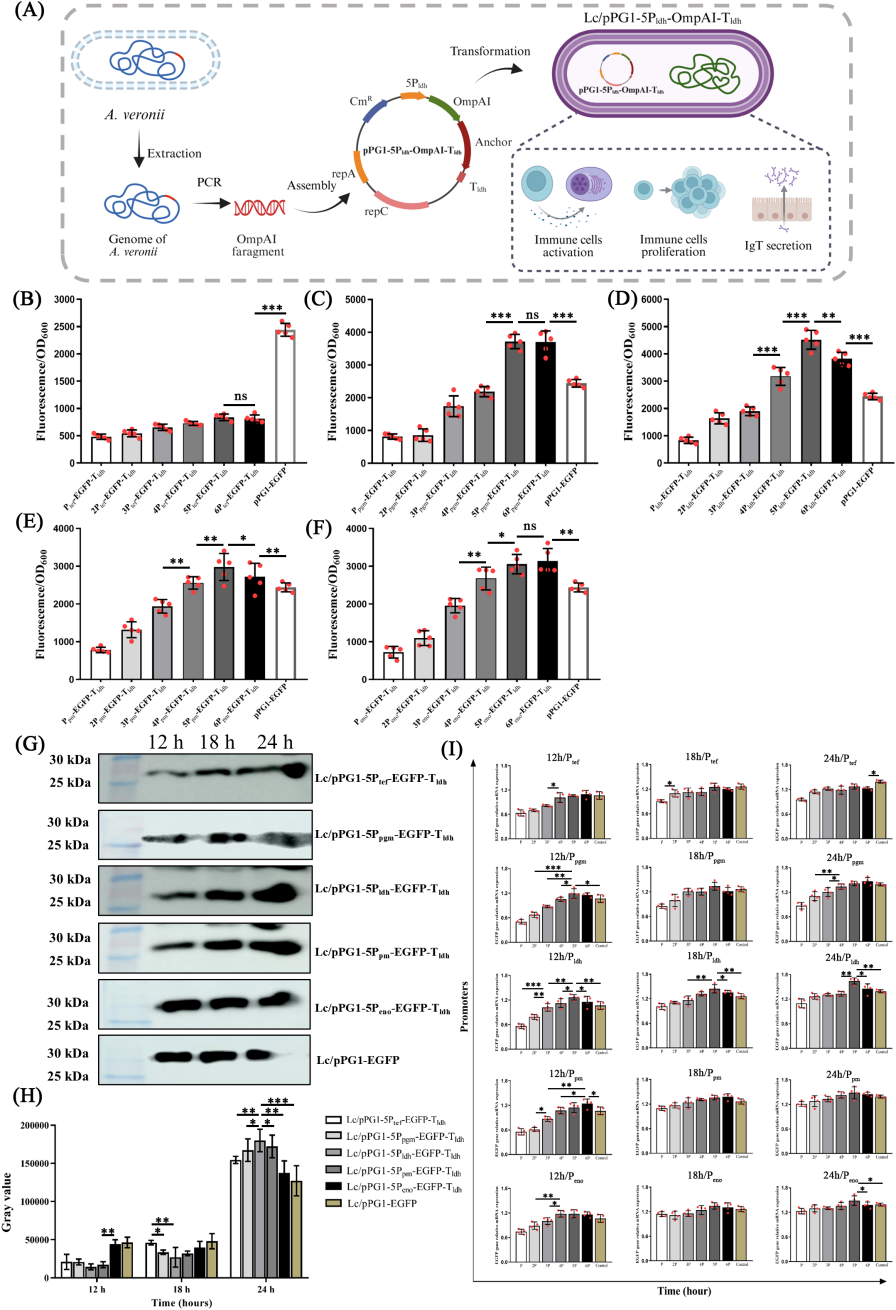
Establishment and characterization of a constitutive expression system in *Lactobacillus casei*. **(A)** Schematic diagram of the construction and induction of mucosal immunity by engineered *L. casei*. **(B–F)** Comparative fluorescence visualization of *L. casei* ATCC393 harboring different promoter-EGFP cassettes: nP_tef_, nP_pgm_, nP_ldh_, nP_pm_, and nP_eno_, respectively, revealing promoter-dependent variations in fluorescence intensity. **(G)** Western blot analysis of EGFP production in engineered strains at distinct cultivation time points, and **(H)** corresponding densitometric quantification of band intensities, demonstrating that nP_ldh_ yielded the highest and most sustained expression levels compared with the alternative promoters. **(I)** RT-PCR validation of EGFP transcription in engineered strains confirmed the constitutive nature of gene expression across all promoter systems. *P < 0.05, **P < 0.01, *** *P* < 0.001.

Genomic analysis of *L. casei* ATCC393 elucidated five candidate promoters (P_tef_, P_pgm_, P_ldh_, P_pm_, P_eno_), derived from highly expressed genes encoding transcription elongation factor GreA, phosphoglycerate mutase, L-lactate dehydrogenase, phosphopentomutase, and phosphopyruvate hydratase, respectively (**Supplementary Figure 1A**). These promoters were PCR-amplified from genomic DNA and fused with the EGFP reporter gene and T_ldh_ terminator, generating nP-EGFP-T_ldh_ expression cassettes. After electroporation of plasmids pPG1-nP-EGFP-T_ldh_ (**Figure 1**) into *L. casei* ATCC393, EGFP expression was validated in bacterial pellets by western blotting and fluorescence observation (**Supplementary Figure 1G**). Fluorescence intensity increased proportionally with promoter copy number in the tandem configurations. Among the constructs tested, the L-lactate dehydrogenase promoter (P_ldh_) yielded the highest fluorescence levels (**Figure 1B**). Time-course analysis (12, 18, 24 h) showed that protein expression and transcription levels for Lc/pPG1-5P_ldh_-EGFP-T_ldh_ peaked at 24 h. Densitometric analysis confirmed that the Lc/pPG1-5P_ldh_-EGFP-T_ldh_ strain produced significantly higher EGFP protein levels than other recombinants after 12 hours (*P* < 0.01, **Figure 1G**), consistent with the transcriptional peak observed at 24 h (**Figure 1**).

### 3.2 pIgR-mediated mucosal IgT immunity identified the hindgut as a non-canonical B cell maturation site

**Figure 2:**
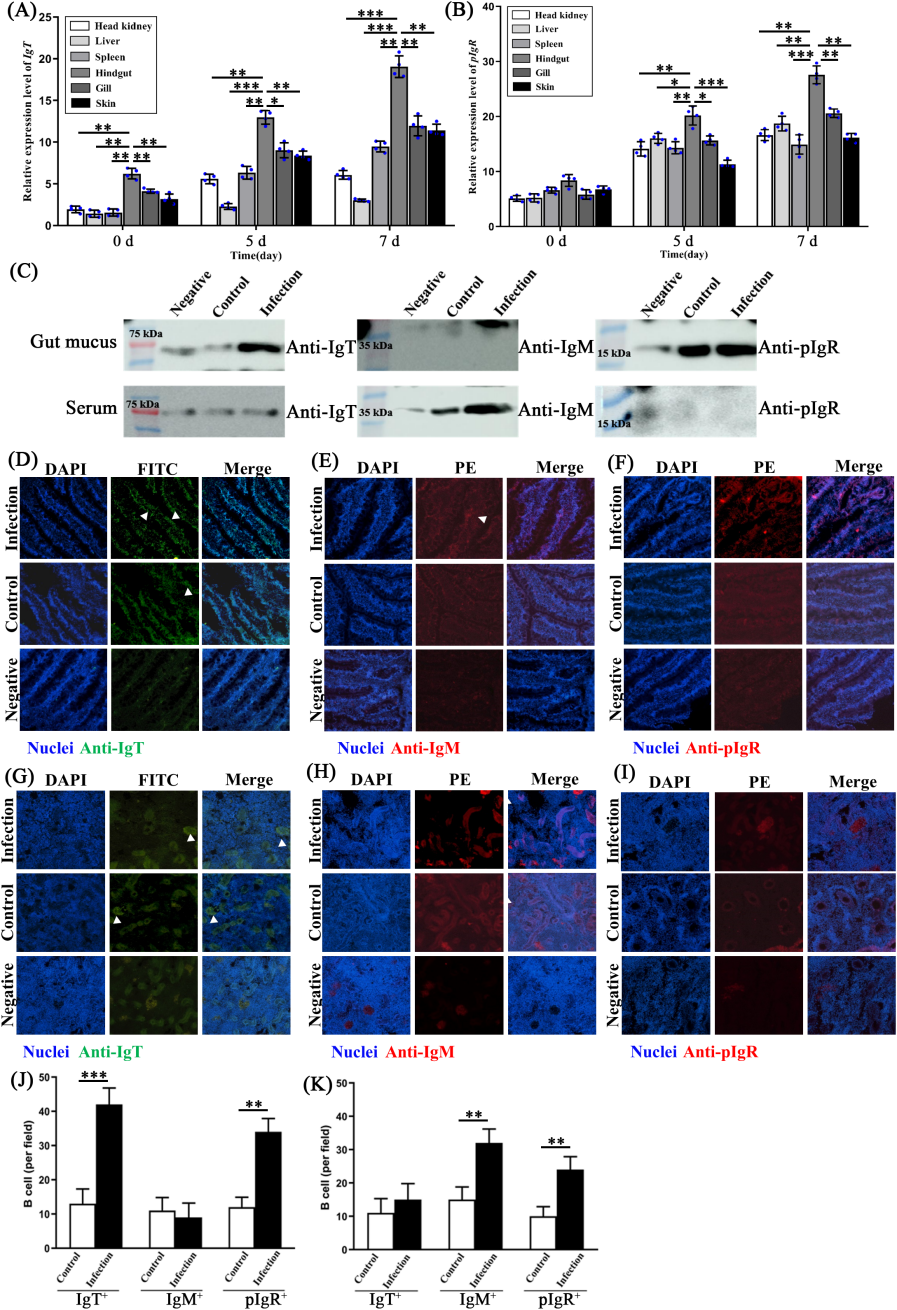
Hindgut as a critical site for IgT^+^ B lymphocyte maturation and mucosal immune activity in *Channa argus* after *Aeromonas veronii* infection. **(A, B)** Quantitative RT-PCR analysis demonstrated that IgT and pIgR transcripts were markedly upregulated in the hindgut compared with other tissues, indicating a preferential mucosal immune activation at this anatomical site upon bacterial challenge. **(C)** Immunoblot assays of gut mucus and serum, probed with anti-IgT, anti-IgM, and anti-pIgR polyclonal antibodies, revealed heightened IgT protein abundance in the intestinal mucus of infected fish, whereas IgM was more prominently detected in serum, suggesting compartmentalized humoral responses. **(D–F)** Immunofluorescence staining of hindgut sections (400 × magnification) clearly localized elevated numbers of IgT^+^, IgM^+^, and pIgR^+^ cells after infection, particularly in the lamina propria and epithelial layers. **(G–I)** The head kidney displayed comparatively lower densities of IgT^+^ and pIgR^+^ cells, consistent with its role as a systemic, rather than mucosal, lymphoid organ. **(J, K)** Quantification from twenty randomly selected microscopic fields confirmed a statistically significant increase in positive cell counts in the hindgut compared to the head kidney. *P < 0.05, **P < 0.01, *** *P* < 0.001.

Transcriptomic analysis of *A. veronii*-infected snakehead hindgut identified immunoglobulin-related differentially expressed genes (DEGs) and mucosal immune pathways (49). Pre-infection, IgT expression in the hindgut was significantly higher than in gill, skin, head kidney, liver, and spleen tissues (*P* < 0.01, **Figure 2**). Post-infection, hindgut IgT transcription markedly increased at 5 days post-infection (*P* < 0.01), with further elevated after challenge. Similar upregulation patterns occurred in gill and skin mucosa (*P* < 0.01), whereas no significant changes were observed in head kidney, liver, or spleen. Baseline pIgR expression showed no significant differences among tissues (*P*>0.05, **Figure 2**), however, infection induced upregulation in all tested tissues, with the highest fold-change observed in the hindgut (*P* < 0.05). Challenge resulted in sustained pIgR elevation in the hindgut (*P* < 0.01).

To preliminarily map the distribution of IgT^+^ and pIgR^+^ cells in *Channa argus*, we developed polyclonal antibodies for detecting these positive cells within tissues and mucus (**Supplementary Figure 2A**). Immunoblotting detected IgT bands (70 kDa) in intestinal mucus and serum from *A. veronii*-infected specimens (7 dpi), while IgM (45 kDa) signals were also present (**Figure 2**). The pIgR protein (20 kDa) was detected exclusively in intestinal mucus. Immunofluorescence analysis showed IgT^+^ cell infiltration within the hindgut lamina propria and epithelium of infected specimens (**Figure 2**). Quantification revealed a 3.23-fold increase in IgT^+^ cells compared to controls (*P* < 0.01, **Figure 2**). IgM^+^ cell numbers in the hindgut showed no significantly change (**Figure 2**). pIgR^+^ cells in infected hindgut epithelia increased 2.83-fold (*P* < 0.01, **Figure 2F**). In the head kidney, IgT^+^ signals were low and showed no intergroup differences (**Figure 2G**). Conversely, head kidney IgM^+^ cells increased 2.13-fold (*P* < 0.01, **Figure 2**), and pIgR^+^ cells clustered around melanomacrophage centers at a 2.4-fold higher density (*P* < 0.01, **Figure 2I**).

### 3.3 Engineered *L. casei* elicited compartmentalized IgT^+^ lymphocyte recruitment and activation kinetics

**Figure 3:**
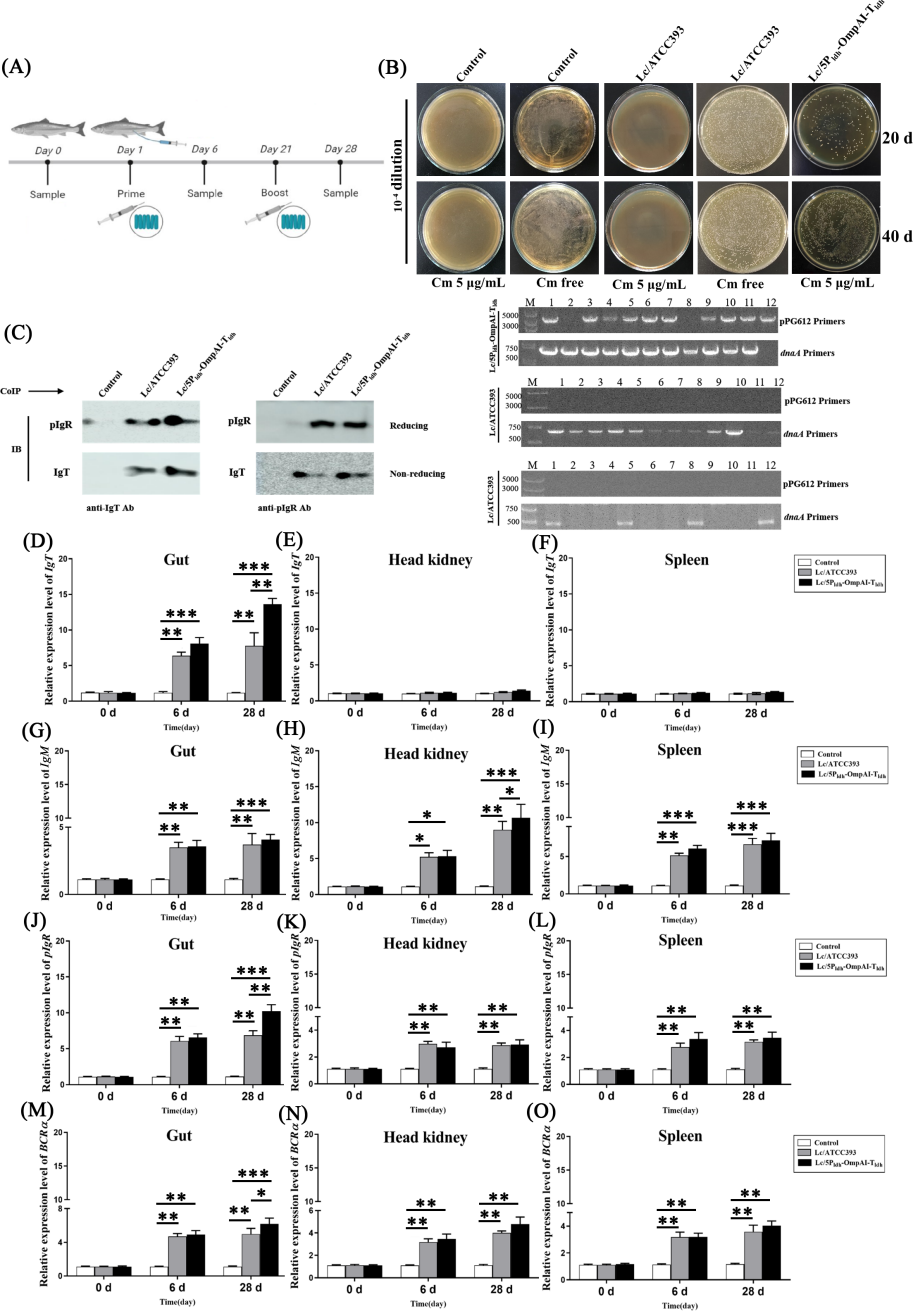
Intestinal persistence of engineered *Lactobacillus casei* and modulation of immune gene expression in *Channa argus*. **(A)** Schematic diagram of the snakehead experimental protocol. **(B)** Quantification of intestinal colonization capacity. Homogenates of the entire intestine from individual fish were cultured on MRS agar, and single colonies were randomly isolated for colony-direct PCR analysis using pPG612-specific primers (upper images) and dnaA-specific primers of *L. casei* (lower images), confirming the persistence of the recombinant strain over the experimental period. **(C)** Co-immunoprecipitation analysis of intestinal mucus demonstrating the interaction between mucosal immunoglobulins and polymeric immunoglobulin receptor (pIgR). Immunoblot assays were performed under both reducing and non-reducing conditions using anti-IgT antibody (left) and anti-pIgR antibody (right), revealing the molecular associations relevant to mucosal immunity. **(D–O)** Tissue-specific transcriptional responses of key immune-related genes after engineered *L. casei* treatment. Quantitative RT-PCR indicated that the mRNA abundance of IgT **(D–F)**, IL-6 **(G–I)**, pIgR **(J–L)**, and BCRα **(M–O)** exhibited significant, tissue-dependent modulation in the hindgut, head kidney, and spleen, respectively. In particular, IgT and pIgR transcripts were markedly upregulated in hindgut tissue, whereas IL-6 and BCRα showed broader modulation across systemic immune organs. *P < 0.05, **P < 0.01, *** *P* < 0.001.

Electroporation of *L. casei* ATCC393 with pPG1-5P_ldh_-OmpAI-T_ldh_ generated engineered strains, as validated by Western blot analysis detecting a 50-kDa OmpAI-specific band (**Supplementary Figure 3**), and surface-localized antigen expression through immunofluorescence (green signal), contrasting sharply with Evans blue-stained, non-fluorescent vector-controls (**Supplementary Figure 3**). After anal intubation (**Figure 3**), the strain colonized the hindgut, maintaining loads of 10^5–^10^6^ CFU/g at day 20 (post-primary) and day 40 (post-booster) (**Table 4**, **Figure 3**). RT-PCR analysis revealed pronounced induction of IgT, IL-6, pIgR, and BCRα transcripts in the hindgut (**Figure 3D**). Besides, elevated expression of IL-6, pIgR, and BCRα was also detected in the head kidney and spleen. Co-immunoprecipitation (Co-IP) assays were performed to examine immunoglobulin interactions. Anti-IgT antibodies precipitated SC-IgT complexes from the intestinal mucus of fish administered either Lc/5P_ldh_-OmpAI-T_ldh_ or control strains. Reciprocally, anti-pIgR antibodies captured IgT across all experimental groups (**Figure 3**). This biochemical evidence confirmed that IgT and pIgR physically bind and form a stable molecular complex *in vivo*, a prerequisite for pIgR-mediated transcytosis.

**Table 4:** Survival of the recombinant *L.casei* in the intestine of fish.

Group/Time	Lc/5P_ldh_-OmpAI-T_ldh_	Lc/ATCC393	Control
	CFU/mL		
20 d	2.12×10^5^	1.08×10^5^	–
40 d	1.24×10^6^	1.19×10^6^	–

**Figure 4:**
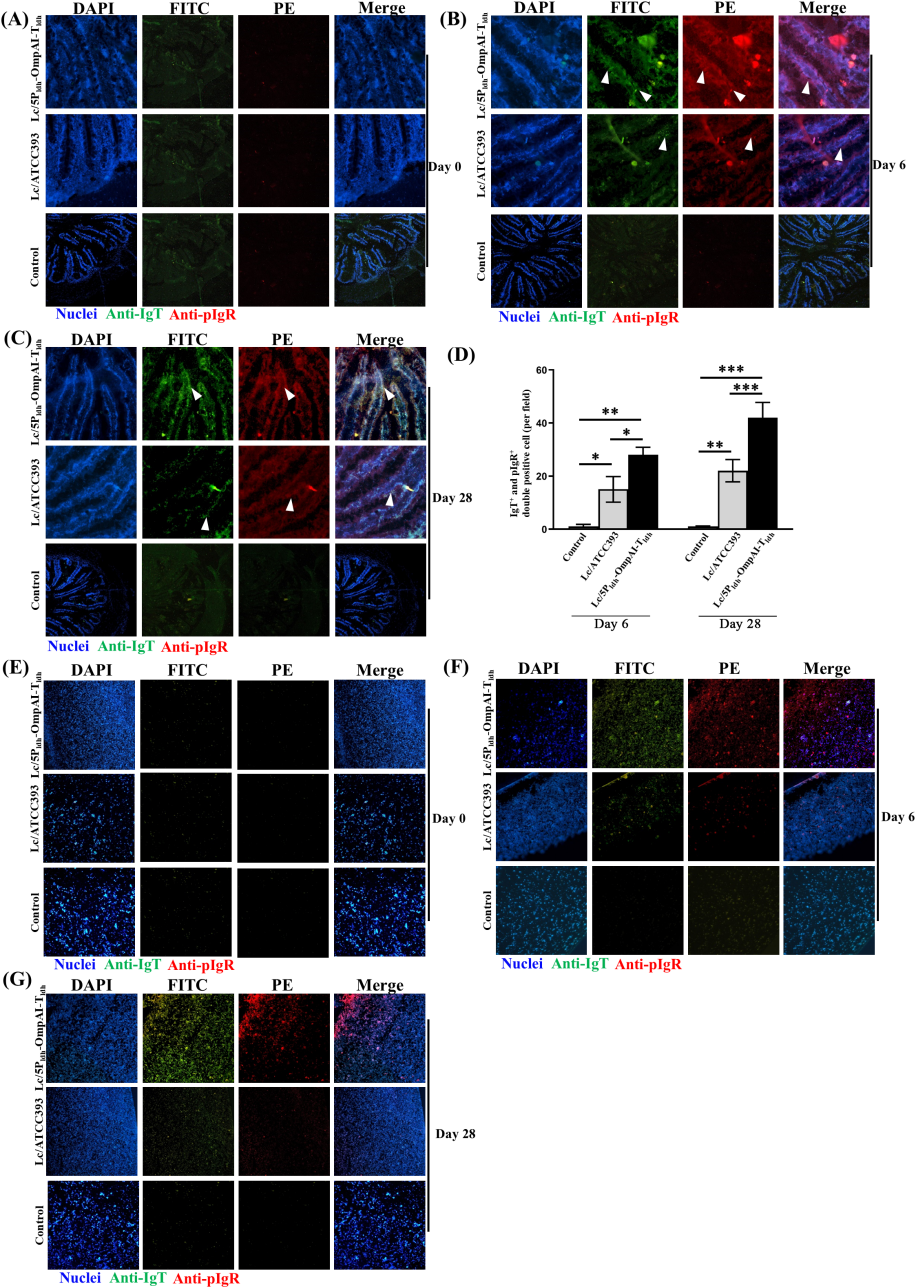
Engineered *Lactobacillus casei* promotes the progressive accumulation of IgT^+^ and pIgR^+^ immune cells in the hindgut of *Channa argus*. **(A–C)** Immunofluorescence analysis revealed distinct spatial distribution patterns of IgT^+^ and pIgR+ cells within posterior intestinal tissues (400×) at 0 d **(A)**, 6 d **(B)**, and 28 d **(C)** post-treatment. **(D)** Quantification of cells demonstrating IgT+/pIgR+ co-localization demonstrated a significant, time-dependent increase at 6 d and 28 d (n=20 fields per section per group, mean ± SEM). **(E–G)** Immunofluorescence characterization of immune cell populations within posterior intestine-associated lymphoid tissue (400×) at 0 d **(E)**, 6 d **(F)**, and 28 d **(G)** post-treatment. *P < 0.05, **P < 0.01, *** *P* < 0.001.

Engineered *L. casei* immunization drove compartmentalized mucosal B-cell recruitment and activation. Immunofluorescence microscopy post-primary immunization detected sparse IgT^+^ and pIgR^+^ double-positive cells within the hindgut. Fish receiving Lc/5P_ldh_-OmpAI-T_ldh_ showed significantly elevated frequencies of these cells compared to controls (**Figures 4A, D**). After the booster immunization, accumulation of IgT^+^ and pIgR^+^ cells increased in the engineered *L. casei* group (**Figure 4**). Analysis of gut-associated leukocytes showed increases in IgT^+^ and pIgR^+^ cells post-primary (**Figure 4E**) and post-booster immunization (**Figure 4**) in the engineered *L. casei* group. Conversely, vector-controls displayed only marginal, adjuvant-driven activation. IgT-dominant, pIgR-facilitated mucosal immunity was conserved across barrier tissues. Immunoblotting confirmed SC-IgT interactions in the intestinal, skin, and gill mucus of booster-immunized Lc/5P_ldh_-OmpAI-T_ldh_ group (**Figure 4**). In contrast, IgM signals remained faint in hindgut and gill mucus and were undetectable in skin mucus, affirming IgT as the dominant mucosal immunoglobulin. Complementary immunofluorescence tracking revealed minimal baseline IgT^+^ and pIgR^+^ leukocytes (**Figure 4**), followed by modest increases post-primary immunization in recombinants (**Figure 4**), culminating in pronounced post-booster expansion, particularly within Lc/5P_ldh_-OmpAI-T_ldh_ hindgut leukocytes. This expansion starkly contrasted with the minimal adjuvant-driven activation observed in controls (**Figure 4**). These findings elucidated an engineered *Lactobacillus*-driven, compartmentalized mucosal immune response characterized by targeted recruitment of IgT^+^ B cells and pIgR-facilitated secretion.

### 3.4 Compartmentalized mucosal IgT^+^ responses and systemic IgM^+^ induction by engineered *L. casei* in teleosts

**Figure 5:**
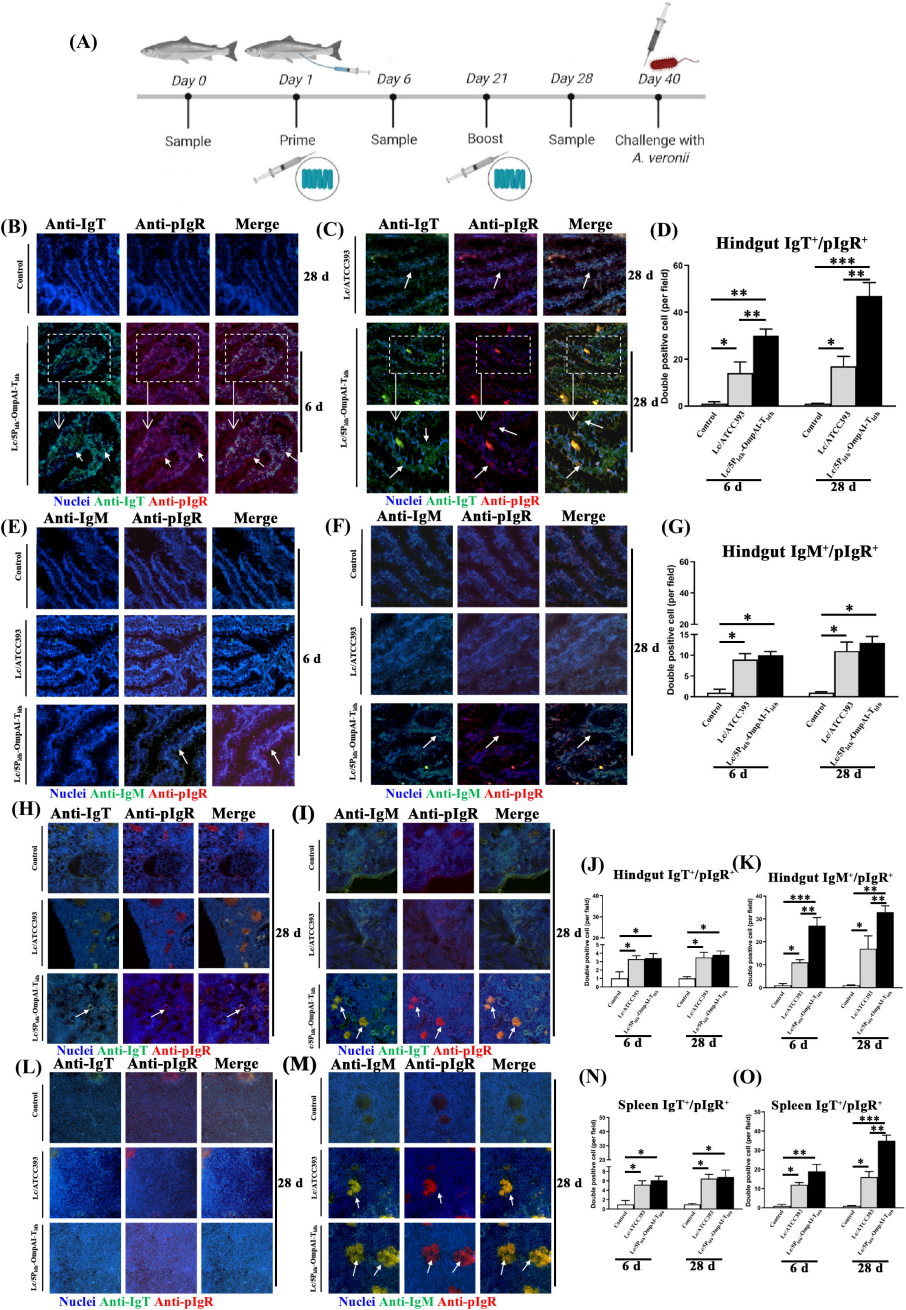
Engineered *Lactobacillus casei* induces significant expansion of IgT^+^/pIgR^+^ double-positive B cell populations in mucosal and systemic immune tissues of snakehead. **(A)** Schematic diagram of the snakehead experimental protocol. **(B, C)** Immunofluorescence micrographs (400×) demonstrating the spatial distribution and density changes of IgT^+^ and pIgR^+^ cells in the posterior intestine following 28 days of probiotic administration, revealing marked mucosal enrichment. **(D)** Quantification of IgT^+^/pIgR^+^ and IgM^+^/pIgR^+^ double-positive cells per 20 microscopic fields, showing statistically significant increases compared to untreated controls. **(E–G)** Representative images and quantitative analysis of IgM^+^ and pIgR^+^ cells in the posterior intestine at day 28, indicating concurrent systemic-type and mucosal-type B cell activation. **(H, I)** Immunolocalization of IgT^+^/pIgR^+^ and IgM^+^/pIgR^+^ populations in the head kidney, demonstrating enhanced systemic immune response in treated fish. **(J, K)** Quantitative enumeration confirming a substantial elevation in double-positive cell (IgT^+^/pIgR^+^ or IgM^+^/pIgR^+^) counts compared with controls. **(L, M)** Distribution of IgT^+^/pIgR^+^ and IgM^+^/pIgR^+^ cells in the spleen at day 28. **(N, O)** Quantitative assessment of splenic double-positive cells per 20 microscopic fields, indicating probiotic-driven enhancement of humoral immune response. *P < 0.05, **P < 0.01, *** *P* < 0.001.

Immunofluorescence analysis of *Lactobacillus*-immunized specimens revealed mucosal compartmentalization. The immunity and challenge of the snakehead fish are shown in **Figure 5**. After primary vaccination, diffuse IgT^+^/pIgR^+^ cells were observed within hindgut epithelia of Lc/5P_ldh_-OmpAI-T_ldh_ group, with signal intensity enhanced post-booster (**Figure 5B**). Minimal fluorescence was detected in vector controls. Quantitative assessment showed that the 47-fold (*P* < 0.01) and 2.76-fold (*P* < 0.01) higher IgT^+^/pIgR^+^ cell densities in the the engineered strain group compared to the control (**Figure 5**). IgM^+^/pIgR^+^ cells were observed in the epithelia, with the engineered *L. casei* group showing increases of 13-fold and 1.18-fold compared to controls (**Figure 5E**). In head kidney, Lc/5P_ldh_-OmpAI-T_ldh_ immunization generated 3.8-fold IgT^+^/pIgR^+^ cells compared to controls (*P* < 0.05) (**Figure 5H**). IgM^+^/pIgR^+^ cells in the head kidney showed 33-fold and 1.94-fold higher densities in the Lc/5P_ldh_-OmpAI-T_ldh_ group (*P* < 0.01, **Figure 5I**). In the spleen, IgT^+^/pIgR^+^ signals were low (**Figure 5L**), while IgM^+^/pIgR^+^ clusters increased 35-fold and 2.19-fold (*P* < 0.01, **Figure 5M**).

### 3.5 Engineered *L. casei* drives IgT-dominated commensal homeostasis across mucosal surfaces

**Figure 6:**
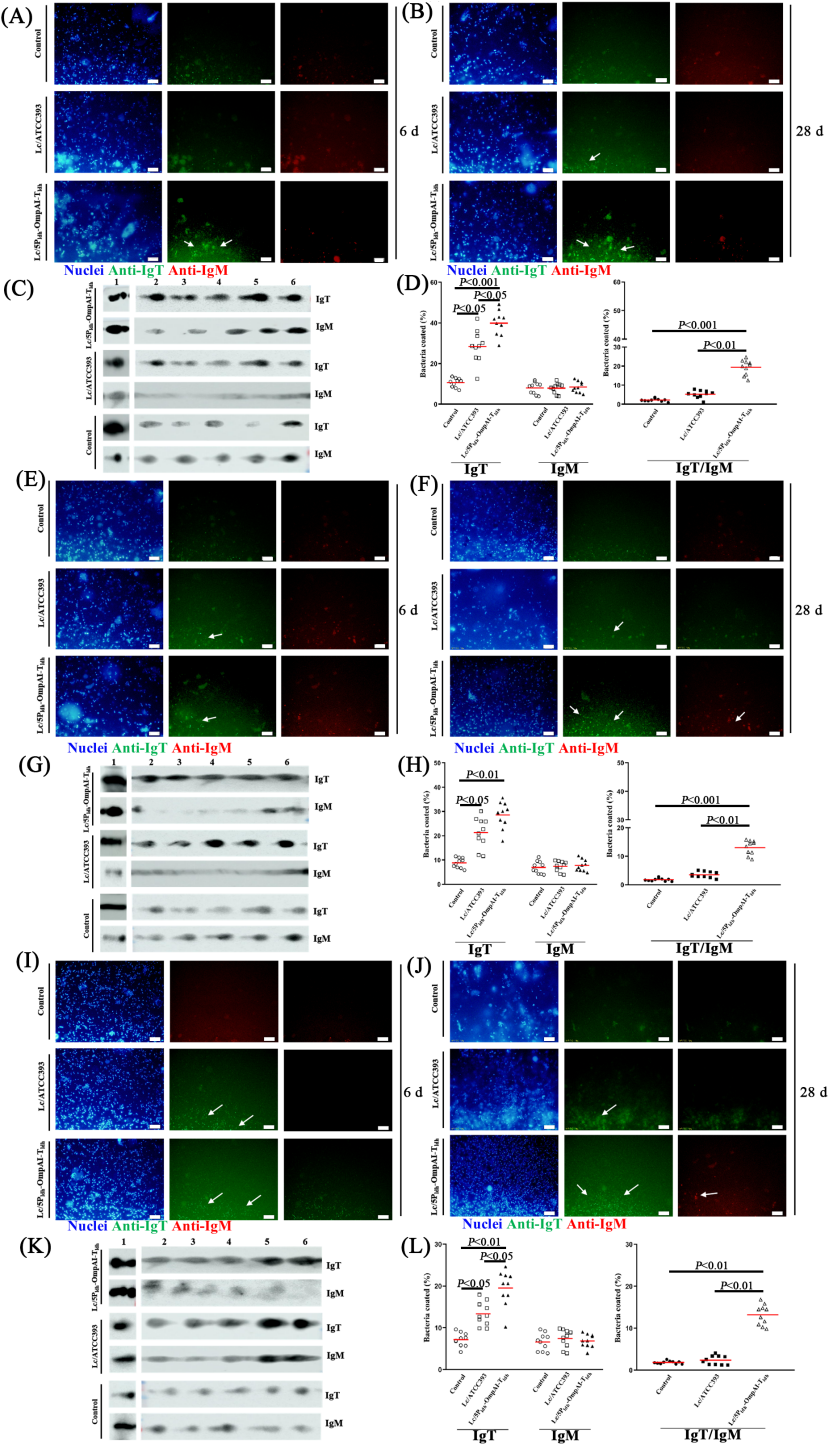
Engineered *Lactobacillus casei* modulates mucosal immune-microbiota interactions by preserving Ig-mediated homeostasis across epithelial sites. **(A, B)** Immunofluorescence detection of Ig-bound bacteria within the posterior intestinal mucosa at 6 d **(A)** and 28 d **(B)** post-treatment (1000×), revealing a time-dependent increase in bacterial coating. **(C)** Western blot analysis confirmed specific Ig binding to mucosa-associated intestinal bacteria at 28 d (Lane 1: Purified IgT/IgM proteins, Lanes 2-6: Representative bacterial isolates, n=5). **(D)** Quantification demonstrated a significant elevation in the proportion of Ig-bound intestinal bacteria at 28 d compared to earlier timepoints. Left: Percentage exhibiting exclusive IgT or IgM binding. Right: Percentage demonstrating concomitant IgT and IgM binding (n=10, mean ± SEM). **(E, F)** Immunofluorescence detection of Ig-bound bacteria within skin mucus at 6 d **(E)** and 28 d **(F)** (1000×), indicating progressive microbial recognition. **(G)** Western blot analysis validated Ig binding specificity for skin mucus-associated bacteria at 28 d (Lane 1: Purified IgT/IgM proteins. Lanes 2-6: Representative isolates, n=5). **(H)** Notably, the proportion of Ig-bound skin bacteria increased markedly by 28 (d) Left: Percentage with exclusive Ig isotype binding. Right: Percentage exhibiting dual IgT/IgM recognition (n=10, mean ± SEM). **(I, J)** Immunofluorescence detection of Ig-bound bacteria within gill mucus at 6 d **(I)** and 28 d **(J)** (1000×), paralleling temporal binding dynamics observed in other mucosal sites. **(K)** Western blot analysis established Ig binding to gill mucus-associated bacteria at 28 d (Lane 1: Purified IgT/IgM proteins. Lanes 2-6: Representative isolates, n=5). **(L)** Quantification revealed a pronounced rise in Ig-bound gill bacteria by 28 (d) Left: Percentage bound solely by IgT or IgM. Right: Percentage demonstrating simultaneous IgT and IgM binding (n=10, mean ± SEM).

Leveraging the dense commensal colonization characteristic of teleost mucosal surfaces, Ig-commensal interactions were investigated in snakehead, a species exhibiting elevated mucosal Ig concentrations. Immunofluorescence analysis at 28 dpi with Lc/5P_ldh_-OmpAI-T_ldh_ revealed a significant predominance of IgT-coated commensals over IgM-bound bacteria within the hindgut, skin, and gill mucus compartments (**Figures 6A, E, F, I, J**). Notably, this observation was corroborated by quantitative immunoblotting (**Figures 6C, K**). Tissue-specific quantification demonstrated markedly elevated IgT-coating frequencies, 40% (hindgut), 29.5% (skin), and 20.6% (gill) of commensals (*P* < 0.05 compared to control groups) (**Figures 6D, L**). These values constituted a substantial increase relative to corresponding IgM-coating frequencies (hindgut: 8.2%, skin: 7.9%, gill: 6.9%). Intriguingly, dual IgT/IgM coating was detected in 20% of hindgut, 13.4% of skin, and 14.5% of gill commensals. These findings elucidated the specialized role of IgT as the principal mucosal Ig isotype engaged in commensal microbiota regulation.

### 3.6 Engineered *L. casei* triggers site-restricted IgT and systemic IgM in teleost

**Figure 7:**
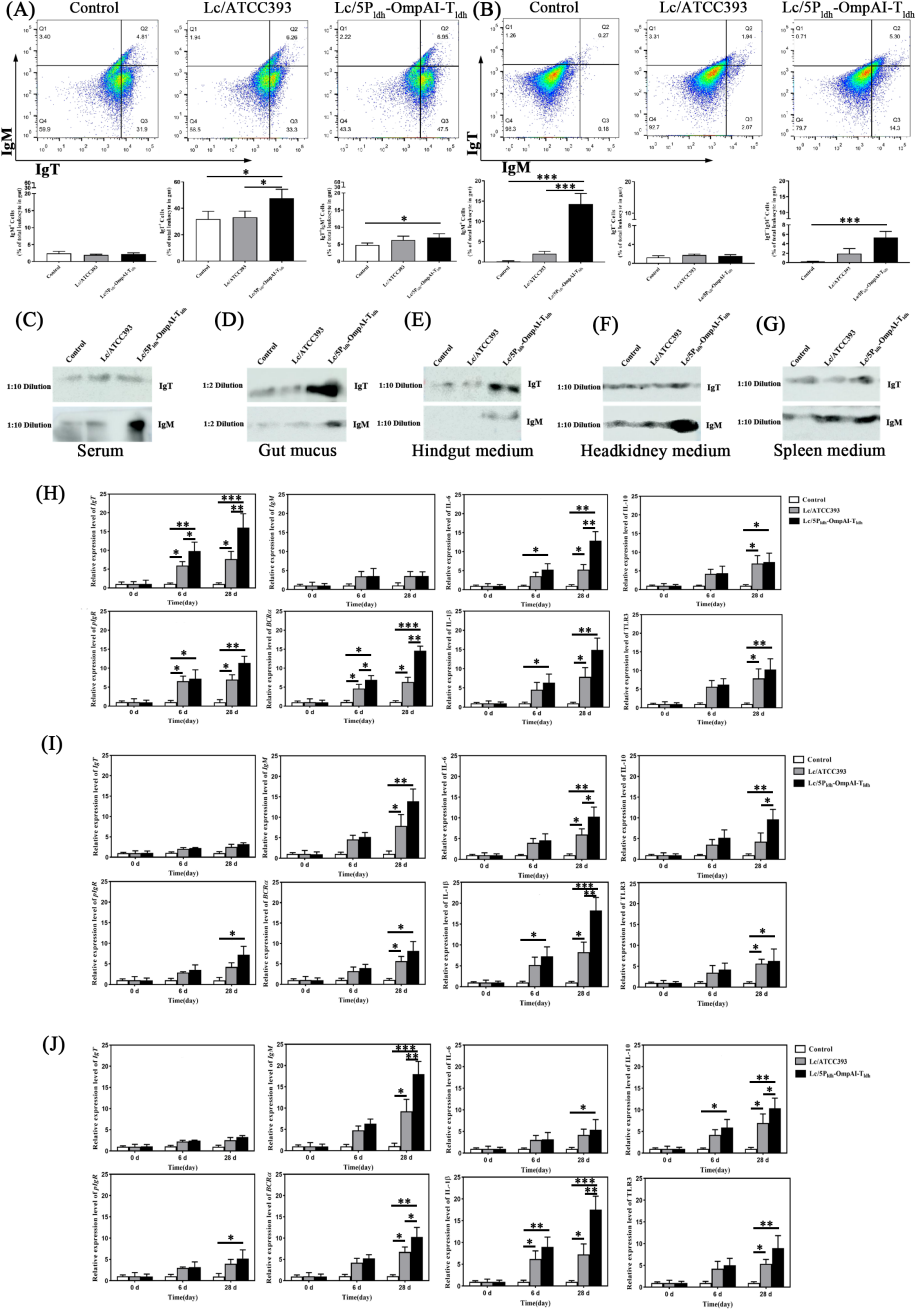
Engineered *Lactobacillus casei* induces compartmentalized immunoglobulin responses in snakehead. **(A, B)** Flow cytometric analysis of IgT+ or IgM+ B cells in total B lymphocytes isolated from hindgut **(A)** and head kidney **(B)** at 28 days post-treatment. **(C–G)** Western blot analyses demonstrating OmpAI-specific IgT was detected in hindgut mucus **(D)** and hindgut culture supernatant **(E)**, OmpAI-specific IgM was observed in serum **(C)**, head kidney **(F)** and spleen **(G)** culture supernatants (Molecular weights indicated in kDa). **(H–J)** Quantitative PCR analysis of immune gene expression in hindgut **(H)**, head kidney **(I)**, and spleen **(J)**. Transcript levels of IgT, IgM, pIgR, BCRα, IL-6, IL-10, IL-1β and TLR3 were significantly upregulated in engineered *L. casei*-treated groups compared to controls. Data represent mean ± SEM (n=8). *P < 0.05, **P < 0.01, *** *P* < 0.001.

Engineered *L. casei* immunization elicited IgT^+^ and IgM^+^ B cell proliferation in hindgut, head kidney, and spleen tissues of snakehead. Flow cytometric analysis revealed that Lc/5P_ldh_-OmpAI-T_ldh_ enhances hindgut IgT^+^ B cell proportions (47.5%, *P* < 0.05), significantly exceeding values in specimens receiving *L. casei* ATCC393 (33.3%, *P* < 0.05) (**Figure 7**). The hindgut IgT^+^/IgM^+^ B cell ratio demonstrated significant elevation in the Lc/5P_ldh_-OmpAI-T_ldh_ group (*P* < 0.05), whereas IgM^+^ B cell proportions remained comparable across groups (*P*>0.05). Head kidney analysis furthermore identified substantial expansion of IgM^+^ B cells (14.3%, *P* < 0.001) (**Figure 7**). Notably, the IgT^+^/IgM^+^ B cell ratio in head kidney was elevated (5.3%, *P* < 0.05 and *P* < 0.001, respectively) despite unaltered IgT^+^ cell counts (*P*>0.05) (**Figure 7**). These data demonstrated preferential mucosal IgT^+^ B cell induction and systemic IgM^+^ B cell expansion by probiotic, indicating tissue-compartmentalized immune activation. Serum analysis detected negligible OmpAI-specific IgT binding across groups at 1:10 dilution. Conversely, specific IgM reactivity was observed exclusively in serum from the Lc/5P_ldh_-OmpAI-T_ldh_ group (**Figure 7**). Intestinal mucus from immunized specimens exhibited dominant IgT-specific reactivity, with IgM detectable only at 1:2 dilution (**Figure 7**). Culture supernatants from immunized tissues reinforced compartmentalization. Hindgut supernatants displayed robust IgT responses exclusively in Lc/5P_ldh_-OmpAI-T_ldh_ group (**Figure 7**). In contrast, head kidney and spleen supernatants contained minimal IgT but displayed modestly elevated IgM reactivity, particularly following Lc/5P_ldh_-OmpAI-T_ldh_ treatment (**Figure 7F**). IgT responses were localized strictly to mucosal sites without systemic distribution.

Antibody titers in serum and intestinal mucus were quantified by ELISA to assess *Lactobacillus*-induced humoral immunity. The mucosal IgT levels in the Lc/5P_ldh_-OmpAI-T_ldh_ group exceeded controls by 2.85-fold and 3.49-fold, respectively, at 28 dpi (*P* < 0.01) (**Supplementary Figure 5**). However, mucosal IgM concentrations remained comparable across groups at lower dilutions (*P*>0.05) (**Supplementary Figure 5**). Serum IgT reactivity demonstrated no significant elevation in *Lactobacillus*-immunized specimens compared to controls (*P*>0.05) (**Supplementary Figure 5**). In contrast, serum IgM titers in the Lc/5P_ldh_-OmpAI-T_ldh_ group surpassed control groups by 2.18-fold and 3.66-fold (*P* < 0.05) (**Supplementary Figure 5**). Dynamic immune responses were further elucidated through RT-PCR quantification of eight immune-related genes (IgT, IgM, pIgR, BCRα, IL-6, IL-10, IL-1β, and TLR3) in hindgut, head kidney, and spleen. Engineered probiotic immunization potentiated immune activation in both mucosal (hindgut, **Figure 7**) and systemic compartments (head kidney/spleen, **Figure 7I**). Primary immunization (day 6) upregulated adaptive immunity genes (IgT, pIgR, BCRα) in the hindgut for *L. casei* groups (**Figure 7**). Notably, post-booster expression of these genes was markedly elevated in Lc/5P_ldh_-OmpAI-T_ldh_ group compared to controls (*P* < 0.01), with hindgut *IgT* transcription exceeding controls levels by 2.15-fold and 16.05-fold. Similarly, the mucosal Ig transporter gene pIgR demonstrated 1.63-fold and 11.36-fold upregulation in engineered *strain* group compared to controls at 28 days post-booster treatment (*P* < 0.01). Hindgut IgM expression, predominantly associated with systemic immunity, remained unaltered across groups (*P*>0.05). Collectively, innate immunity genes (IL-6, IL-10, IL-1β, TLR3) showed differential upregulation after probiotic treatment (**Figure 7**). Adaptive immune markers peaked post-primary immunization, whereas innate genes sustained elevated expression post-booster within the hindgut mucosa. In head kidney tissue, IgT transcription remained comparable across groups post-immunization (*P*>0.05). IgM expression was significantly elevated in Lc/5P_ldh_-OmpAI-T_ldh_ group compared to controls, and sustained 1.76-fold and 13.89-fold upregulation post-booster treatment (*P* < 0.01). Innate immunity genes (IL-6, IL-10, IL-1β, TLR3) demonstrated kinetic parallels with hindgut observations, exhibiting rapid transcriptional amplification after booster administration. Notably, IL-1β expression in engineered strain group exceeded control levels by 28 days (*P* < 0.001) (**Figure 7**). Conversely, spleen tissue representing systemic immunity, displayed no significant modulation of mucosal immunity markers (IgT, pIgR). IgM expression patterns were consistent with head kidney findings, with the engineered *L. casei* group demonstrating 1.94-fold and 17.98-fold elevation post-secondary immunization versus comparators (*P* < 0.01), indicating progressive enhancement of systemic humoral responses. Innate immunity mediators (IL-6, IL-10, IL-1β, TLR3) manifested successive upregulation with booster immunization (**Figure 7**), consistent with coincidental activation of intestinal mucosa and systemic immune response.

### 3.7 Engineered *L. casei* confers cross-tissue mucosal protection against *A. veronii* challenge

**Figure 8:**
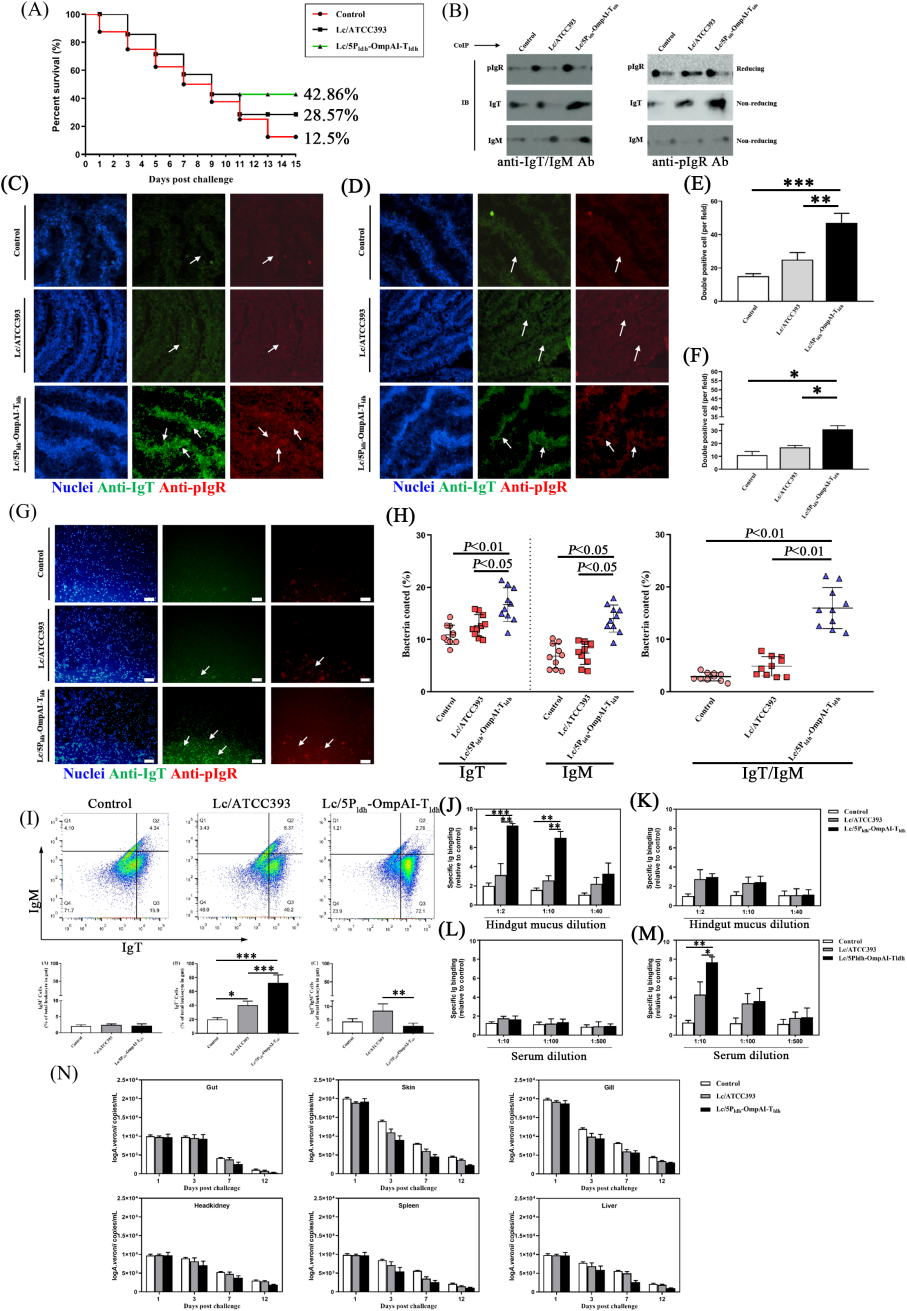
Engineered *Lactobacillus casei* confers protection against *A. veronii* infection in snakehead. **(A)** Survival kinetics post-challenge with *A. veronii*. Engineered *L. casei*-immunized groups exhibited significantly enhanced survival rates compared to controls. **(B)** Co-immunoprecipitation of gut mucus immunoglobulins from survivor fish, demonstrating pathogen-specific binding. **(C–F)** Immunofluorescence localization in hindgut tissues of IgT^+^ and pIgR^+^ **(C)** and IgM^+^ and pIgR^+^ **(D)** dual-positive cells (white arrows). Quantification of dual-positive cells per 20 fields **(E, F)** revealed significant increases. **(G)** Bacterial immunoglobulin coating in gut mucus. Representative differential interference contrast (DIC) images with DAPI (blue), anti-IgT (green), and anti-IgM (red) staining (1000×). **(H)** Quantification of gut mucus bacteria coated with IgT or IgM (left) or coated with IgT and IgM (right) in survivor fish (n=10). **(I)** Flow cytometric analysis of IgT^+^/IgM^+^ B cell propotions in hindgut were elevated in engineered *L. casei* group. **(J–M)** Antigen-specific antibody binding to *A. veronii* of survived fish. Serial dilutions of gut mucus **(J, K)** and serum **(L, M)** from survivor fish exhibited potent IgT reactivity to *A. veronii* OmpAI compared to IgM. **(N)** *A. veronii* burdens in systemic organs (spleen, liver, kidney) were reduced in probiotic groups from survivor fish. Data represent as mean ± SEM (n=10). *P < 0.05, **P < 0.01, *** *P* < 0.001.

To evaluate immunoprotective efficacy, an *A. veronii* immersion challenge was conducted, with initial mortalities recorded within 3 days post-infection. While persistent mortality characterized the control group throughout the second week, the Lc/5P_ldh_-OmpAI-T_ldh_ group demonstrated significantly stabilized survival rate at 42.86% (**Figure 8**). In contrast, sustained lethality persisted within the *L. casei* ATCC393 and control groups, resulting in restricted survival rates of 28.5% and 12.5%, respectively. Based on these mortality data (57.1% mortality in vaccinated and 87.5% in control), the Relative Percent Survival (RPS) was calculated as 34.7%. To determine whether the observed protection was driven by specific mucosal adaptive immunity, we restricted our immunological analyses (IgT/IgM titers, pIgR expression, and bacterial load) to the survivor fish at the end of the 14-day challenge period (**Figure 8B**). The mucosal immunity was assessed by quantifying immunoglobulin (IgT/IgM) and polymeric immunoglobulin receptor (pIgR) abundance in intestinal mucus after challenged. Markedly elevated IgT and pIgR protein levels were identified in the Lc/5P_ldh_-OmpAI-T_ldh_ group by immunoblot assays (**Figure 8**), implicating lymphocytic IgT secretion coupled with pIgR-mediated transcytosis. Conversely, minimal IgT and IgM production characterized the *L. casei* ATCC393 and control groups (**Figure 8**), potentially reflecting pathogen-associated suppression of epithelial or antigen-presenting cell function. To elucidate cellular distribution, hindgut tissues were examined 14 days post-infection. Extensive proliferation of IgT^+^ and pIgR^+^ cells was visualized in the engineered strain-treated group (**Figure 8**). Quantitative analysis confirmed a statistically significant expansion of IgT^+^ and pIgR^+^ double-positive populations in the engineered probiotic group relative to the control group (*P* < 0.01, **Figure 8**). Analogously, intense co-localization of IgM^+^ and pIgR^+^ signals was observed within the Lc/5P_ldh_-OmpAI-T_ldh_ group (**Figure 8**). Statistical evaluation substantiated a significant enrichment of IgM^+^ and pIgR^+^ cells in the engineered *L. casei* group (*P* < 0.05, **Figure 8**).

Lc/5P_ldh_-OmpAI-T_ldh_ administration elicited significantly elevated IgT- and IgM-binding frequencies on mucus-associated bacteria compared with the survivor fish controls (*P* < 0.05, **Figure 8G**). Quantitative analysis revealed that 15% of the intestinal microbiota within the engineered strain group presented a double-positive coating, a frequency surpassing the *L. casei* ATCC393 and control groups (*P* < 0.01, **Figure 8**). The Lc/5P_ldh_-OmpAI-T_ldh_ treatment induced a dominant IgT^+^ B cell population, constituting 72.1% of total hindgut B lymphocytes in survivor fish, a proportion significantly exceeding control values (*P* < 0.001, **Figure 8**). Conversely, the IgT^+^/IgM^+^ ratio manifested a marked reduction relative to the *L. casei* ATCC393 group (*P* < 0.01), whereas IgM^+^ B cell proportions remained statistically constant across all experimental groups (*P*>0.05). Intestinal mucus derived from the engineered strain-treated survivor fish maintained high specific IgT titers detectable at ≥10-fold dilution, levels significantly elevated controls (*P* < 0.01, **Figure 8**). Parallel assessment of systemic responses revealed that serum-specific IgM concentrations were upregulated in Lc/5P_ldh_-OmpAI-T_ldh_ survivor fish (*P* < 0.01, **Figure 8**). Serum IgT levels manifested no significant fluctuations in survivor fish across the examined groups (**Figure 8**). Post-challenge intestinal mucus IgM levels increased compared to the post-booster phase (*P*>0.05, **Figure 8**), indicating a generalized stress response distinct from the specific IgT elevation. *A. veronii* loads in diverse tissues were quantified at 1, 3, 7, and 12 dpi to evaluate colonization kinetics. Bacterial loads within *Lactobacillus*-treated groups remained significantly lower than the unvaccinated control during the initial 7 dpi, elucidating a inhibitory effect during the acute infection phase (**Figure 8**). A rapid reduction in pathogen density within the skin and gills was observed by 3 dpi in the Lc/5P_ldh_-OmpAI-T_ldh_ group, while significant attenuation in the hindgut and systemic organs occurred by 5 dpi (*P* < 0.01). The magnitude of bacterial clearance in the engineered strain group surpassed the wild-type *L. casei* group, confirming enhanced protective efficacy conferred by heterologous antigen expression.

### 3.8 Engineered *L. casei* regulates the gut microbiome

**Figure 9:**
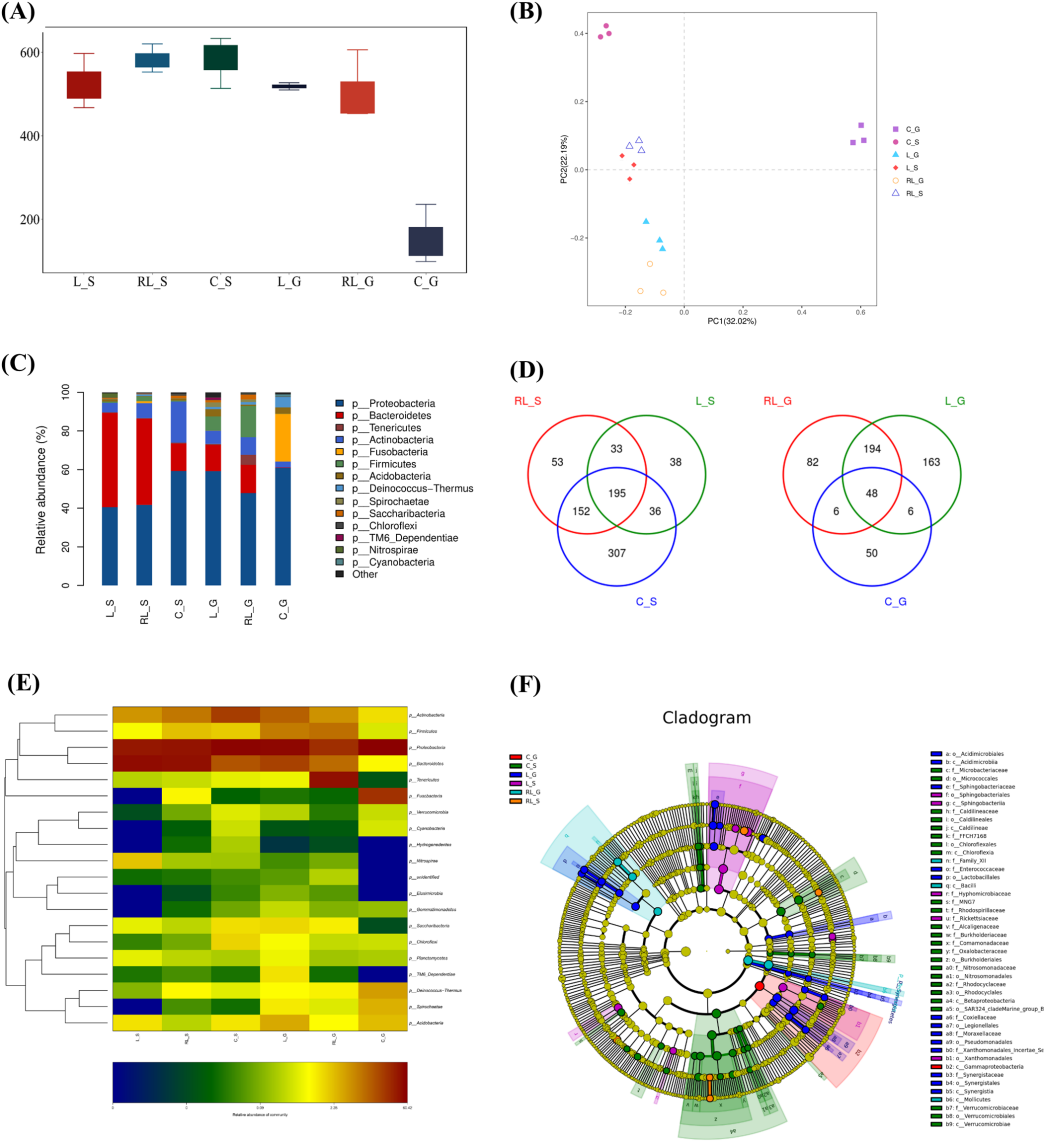
Regulation of gut microbiota in response to *A. veronii* challenge. Analysis of alpha **(A)** and beta **(B)** diversity. **(C)** Phylum-level compositional shifts. **(D)** OTU distribution across experimental groups. **(E)** Heatmap of dominant phyla in hindgut. **(F)** LEfSe cladogram of differentially enriched taxa.

Skin mucus and hindgut content samples were collected from *C. argus* at 28 days post-immunization. Samples from the *L. casei* group were denoted as L_S (skin mucus) and L_G (hindgut contents), the Lc/5P_ldh_-OmpAI-T_ldh_ group as RL_S and RL_G, and control samples as C_S and C_G. Total RNA was isolated from all specimens for 16S rRNA gene sequencing. Alpha diversity indices indicated elevated microbial richness in hindgut contents of both *L. casei*- and Lc/5P_ldh_-OmpAI-T_ldh_-treated fish compared to controls (**Figure 9**). Beta diversity, assessed via Bray-Curtis dissimilarity metrics derived from amplicon sequence variant (ASV) abundance profiles, was visualized through principal coordinates analysis (PCoA). In skin mucus, distinct clustering separated control specimens (C_S) from *Lactobacillus*-exposed groups (L_S, RL_S) along PC1, which accounted for 32.02% of variance, with controls positioned in the positive quadrant and treated samples in the negative quadrant (**Figure 9**). Hindgut contents similarly displayed segregation along PC2 (22.19% variance explained), wherein controls (C_G) occupied the positive quadrant while treated groups (L_G, RL_G) clustered proximal to the origin (**Figure 9**). These patterns elucidated a profound reconfiguration of microbial community architecture in both skin mucus and hindgut microbiota following *Lactobacillus* administration.

At the phylum level, relative abundances in skin mucus and hindgut contents are depicted in **Figure 9**. *Bacteroidetes* proportions were markedly augmented in skin mucus of treated groups compared to controls, whereas *Proteobacteria* and Actinobacteria levels declined concomitantly (**Figure 9**). In hindgut contents, *Firmicutes* and *Bacteroidetes* abundances rose substantially, offset by diminished *Fusobacteria* representation (**Figure 9**). Venn diagrams delineated overlapping and distinctive operational taxonomic units (OTUs) across groups, underscoring intersample variability in microbial taxa (**Figure 9**). Heatmap profiling corroborated pronounced *Firmicutes* enrichment within hindgut communities of both *L. casei*- and Lc/5P_ldh_-OmpAI-T_ldh_-treated cohorts (**Figure 9**). Linear discriminant analysis effect size (LEfSe) was employed to pinpoint taxa differentially prevalent in skin mucus and hindgut microbiota. The resultant cladogram, constructed by hierarchically mapping taxa with linear discriminant analysis scores exceeding 2.0 (*P* < 0.05), illuminated divergent enrichment signatures (**Figure 9**). Notably, *Sphingobacteriaceae* (clade e) and *Sphingobacteriales* (clade f) predominated in *L. casei*-treated samples (L_S, L_G). In contrast, *Comamonadaceae* (clade x) prevailed in skin mucus of the Lc/5P_ldh_-OmpAI-T_ldh_ group (RL_S), while Family_XII (clade n) was accentuated in hindgut counterpart (RL_G).

## 4 Discussion

Aquatic infections caused by *A. veronii* have emerged as a significant threat to both global aquaculture and public health, with potential zoonotic transmission to humans (3). As a mucosal pathogen, *A. veronii* primarily invades through epithelial surfaces such as the intestine, skin, and gills, which constitute critical portals while functioning as inductive sites for localized immunity (50). Hence, the development of mucosal vaccines capable of inducing robust local and systemic protection represents an essential priority in aquaculture immunoprophylaxis. However, to date, investigations into efficacious mucosal vaccination strategies against aquatic bacterial pathogens remain limited.

Probiotic-based antigen delivery systems have gained increasing attention due to dual function in promoting host health and facilitating immune activation (51). Specifically, *Lactobacillus* spp. possess inherent advantages in mucosal colonization and immune modulation (15, 60). In the present study, an engineered *L. casei* strain was developed utilizing a quintuple tandem repeat of the *ldh* promoter to drive the expression of the outer membrane protein OmpAI from *A. veronii.* This design significantly enhanced transcriptional efficiency and protein yield, attributed to increased mRNA stability and translational competency. The expression system thereby circumvented the need for exogenous protein purification, aligning with scalable, cost-effective vaccine production strategies. Prior studies in mammals have demonstrated that *Lactobacillus* can be recognized by mucosal antigen-presenting cells (APCs) and initiate downstream adaptive immune cascades (52–55). Although mechanistic data in fish remain sparse, the present findings provided compelling *in vivo* evidence that *L. casei* engages gut-associated lymphoid tissue (GALT), resulting in the activation of B cells and local cytokine secretion. Pathogen engagement of the GALT initiates internalization by professional APCs, predominantly macrophages and dendritic cells, for processing within the phagolysosomal compartment. OmpAI epitopes expressed by *L. casei* are sequestered onto Major Histocompatibility Complex (MHC) Class II molecules for presentation to CD4^+^ T helper cells, a mechanism fundamental to orchestrated B cell differentiation and immunoglobulin isotype switching. Although direct proteomic quantification of MHC molecules remains constrained by a paucity of species-specific antibodies for *C. argus*, transcriptional results elucidated upregulation of IL-1β and TLR3 within the hindgut (**Figure 7**). Elevation of IL-1β, a definitive indicator of APC activation, alongside the pattern recognition receptor TLR3, substantiates the activation of innate recognition cascades that license efficient antigen presentation through the MHC pathway.

**Figure 10:**
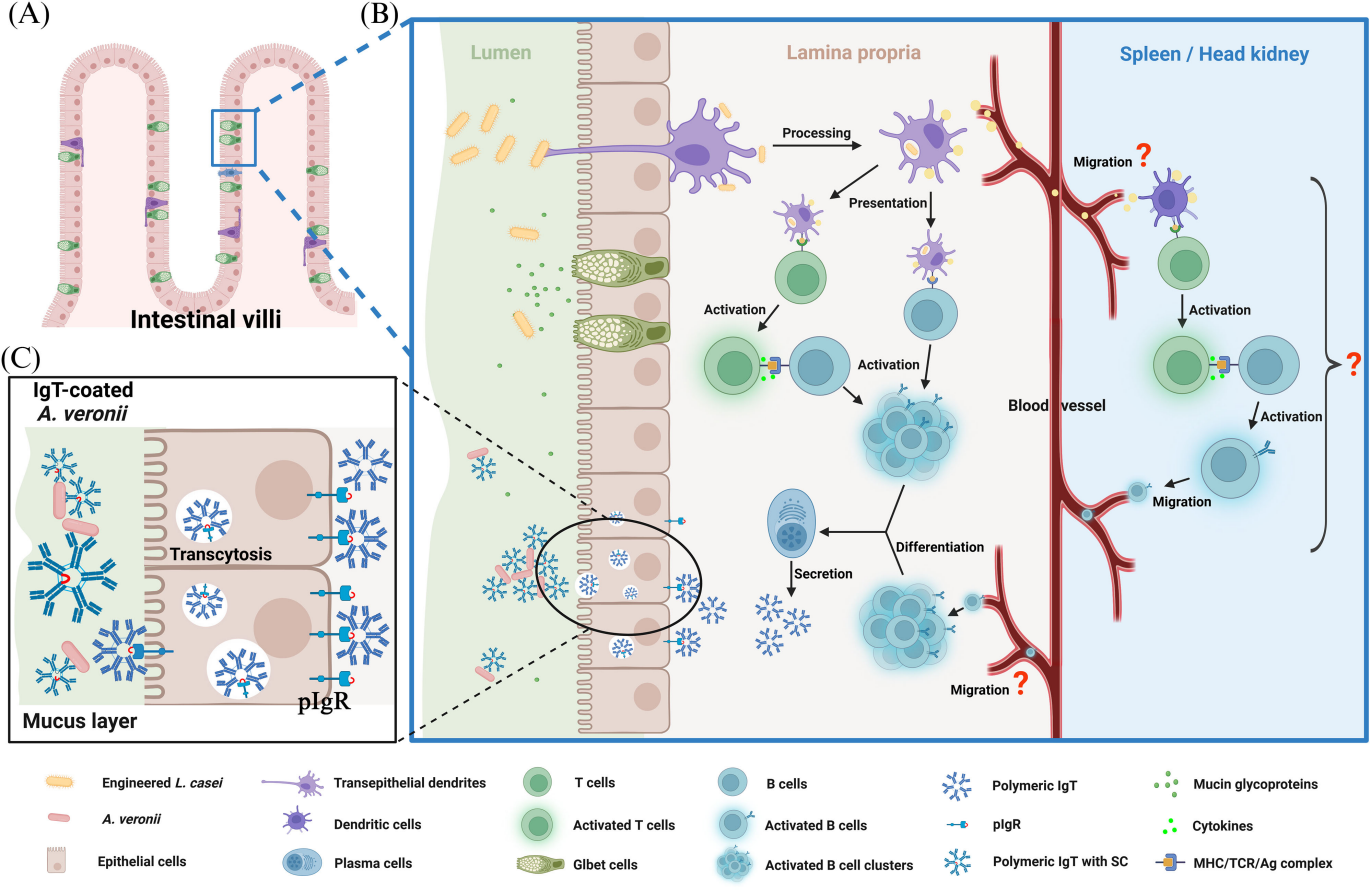
Schematic diagram illustrating the Engineered *Lactobacillus casei*-mediated protective mechanisms against *Aeromonas* species. The engineered *Lactobacillus casei* induced compartmentalized mucosal immunity in *Channa argus, and* enhanced pathogen clearance, elevated survival rates upon challenge, and modulated commensal microbiota composition.

Notably, administration of the engineered strain led to pronounced transcriptional upregulation of immune-related genes including IgT, IL-6, pIgR, and BCRα within the intestinal mucosa, strongly suggesting mucosal B cell activation and epithelial immune engagement. Besides, elevated IL-6 expression may reflect activation of DCs, in line with mammalian models wherein microbial internalization through TLR4 signaling induces IL-6 and IL-10 release, potentiating IgA class switching (56, 57). Although direct quantification of BAFF and APRIL was limited by reagent availability, the significant upregulation of IL-6 and IL-10 genes observed in the GALT suggested a cytokine milieu conducive to IgT^+^ B-cell proliferation. This profile mirrors BAFF/APRIL-mediated pathways described in model teleosts, suggesting a conserved mechanism of mucosal B-cell activation. Analogously, the intestinal immune architecture of teleosts, despite lacking Peyer’s patches, appears capable of sustaining localized IgT responses. The elucidation of functional causality between pIgR expression and mucosal immunoglobulin secretion represents a critical question in teleost immunology. Biochemical analyses conducted herein bridge the conceptual gap between transcriptional correlation and functional causation. As pIgR necessitates basolateral expression and transcytosis prior to apical cleavage, the detection of stable IgT-SC complexes within the lumen confirms the active transport of IgT across the epithelial barrier. Such results corroborate the transport mechanisms established in *Oncorhynchus mykiss*, validating the IgT-pIgR axis as the definitive mechanistic driver of mucosal protection rather than a mere correlative phenomenon. Furthermore, the expression pattern of pIgR, predominantly within epithelial cells, and the co-localization with IgT in the mucus layer, substantiates the hypothesis that teleost pIgR mediates transcytosis of IgT into mucosal secretions, functionally analogous to the sIgA-pIgR system in mammals (58). Moreover, immunofluorescence analysis revealed a marked accumulation of IgT^+^ cells in both the lamina propria and mucosal epithelium after anal intubation with probiotic, confirming that the distal intestine represents a key inductive site for IgT-mediated responses in *C. argus* (**Figure 10**). Approximately 40% of mucosa-associated bacteria were coated with IgT, mirroring reports in *Oncorhynchus mykiss*, and emphasizing the conserved role of IgT in maintaining microbial homeostasis (39). Interestingly, OmpAI, as a protein antigen, may induce class switching through a T cell-dependent (TD)-like mechanism, although the contribution of BAFF/APRIL signaling in this context requires further investigation. The presence of IgM-bound microbes in control fish highlighted the isotype-specific affinity of immunoglobulins to commensals in teleosts. Immunization with the engineered *L. casei* reshaped the skin mucus and hindgut microbiota of *C. argus*. At the phylum level, engineered *L. casei* treatment enhanced *Bacteroidota* abundance and reduced *Proteobacteria* and *Actinobacteriota* in the skin, while significantly increasing *Firmicutes* and *Bacteroidota* in the hindgut. The observed microbial reconfiguration is hypothesized to exert a synergistic protective effect in alignment with antigen-specific immunity. The proliferation of *Firmicutes* underpins the biosynthesis of short-chain fatty acids (SCFAs), particularly butyrate, providing metabolic substrates for enterocytes while simultaneously fortifying epithelial barrier integrity through the induction of antimicrobial peptide expression. The resulting SCFA-mediated mechanisms establish a permissive milieu for immunological homeostasis. Furthermore, competitive exclusion is facilitated through probiotic engraftment, wherein the physical occupation of mucosal attachment loci and sequestration of environmental nutrients by the enriched beneficial flora, characterized by the augmentation of *Firmicutes* and the attenuation of *Proteobacteria*, constrain the colonization capacity of *A. veronii*. The commensal community functions as an innate biological bastion, attenuating the primary pathogen burden and facilitating the specific IgT-mediated clearance of the infectious agent.

Our data demonstrated that mucosal delivery of the engineered *L. casei* strain successfully elicited a compartmentalized immune response characterized by IgT dominance at the mucosal interface and IgM prevalence in systemic lymphoid organs such as the spleen and head kidney. This spatial distinction underscores the specialized roles of teleost immunoglobulin isotypes in host defense. It is worth noting that although IgT titers were comparatively lower than those observed in trout, this discrepancy may reflect interspecies immunological variability rather than limitations of vaccine efficacy. Furthermore, concurrent upregulation of innate (IL-6, IL-10, TLR3) and adaptive (IgT, IgM) markers reinforces the notion of integrated immune activation encompassing both cellular arms. Immunized fish exhibited a relative percent survival of 42.86% upon immersion challenge, alongside significant reductions in bacterial burden and mortality rate. These findings indicated that the engineered probiotic effectively enhanced both mucosal and systemic immunity, potentially through synergistic modulation of epithelial barriers, APC function, and B cell differentiation. Prior studies have established that *Lactobacillus* can activate macrophages and DCs to produce IL-12, subsequently promoting Th1 polarization and cytotoxic lymphocyte responses (59). The current results extend this paradigm to fish mucosal immunity, highlighting the translational potential of engineered probiotics in aquatic vaccine development. To commercialize this hindgut IgT-pIgR axis paradigm, future efforts must replace impractical anal delivery with acid-shielded oral microencapsulation, integrate auxotrophic containment for GMM biosafety, and validate mechanism conservation across diverse teleosts. Resolving these technical, regulatory, and comparative immunology challenges is paramount for translating these mechanistic insights into scalable, field-ready mucosal vaccines for global aquaculture.

## 5 Conclusion

This study established a strong proof-of-concept for a targeted mucosal vaccination strategy in *Channa argus*. We demonstrated that the administration of engineered *L. casei* expressing *A. veronii* OmpAI effectively leverages the hindgut IgT-pIgR axis, a critical functional hub for immune induction in this species. This targeted activation resulted in the compartmentalized recruitment of IgT^+^ B cells, pIgR-mediated antibody transcytosis, and specific microbiota modulation, ultimately conferring significant protection against *A. veronii*. While these findings validate the feasibility of stimulating mucosal immunity via the hindgut, the translation of this strategy to field-level aquaculture will require further optimization of oral delivery systems, such as microencapsulation, to ensure stability and biosafety. This work provides a valuable theoretical framework and a technological prototype for developing next-generation, non-invasive immunoprophylactics tailored to the unique mucosal landscape of teleost fish.

## Data Availability

The data presented in the study are deposited in the NCBI Sequence Read Archive (SRA) repository, accession number PRJNA1425734.
