# Supplementary material for: Engineered Lactobacillus casei targets the IgT-pIgR axis to confer mucosal protection against Aeromonas veronii in snakehead (Channa argus)
Source: Front Immunol. 2026 Mar 23;17:1759765. doi: 10.3389/fimmu.2026.1759765 (PMC13050950; doi:10.3389/fimmu.2026.1759765)
Supplement: Supplementary file 1 [file DataSheet1.docx]

**Supplementary Figures
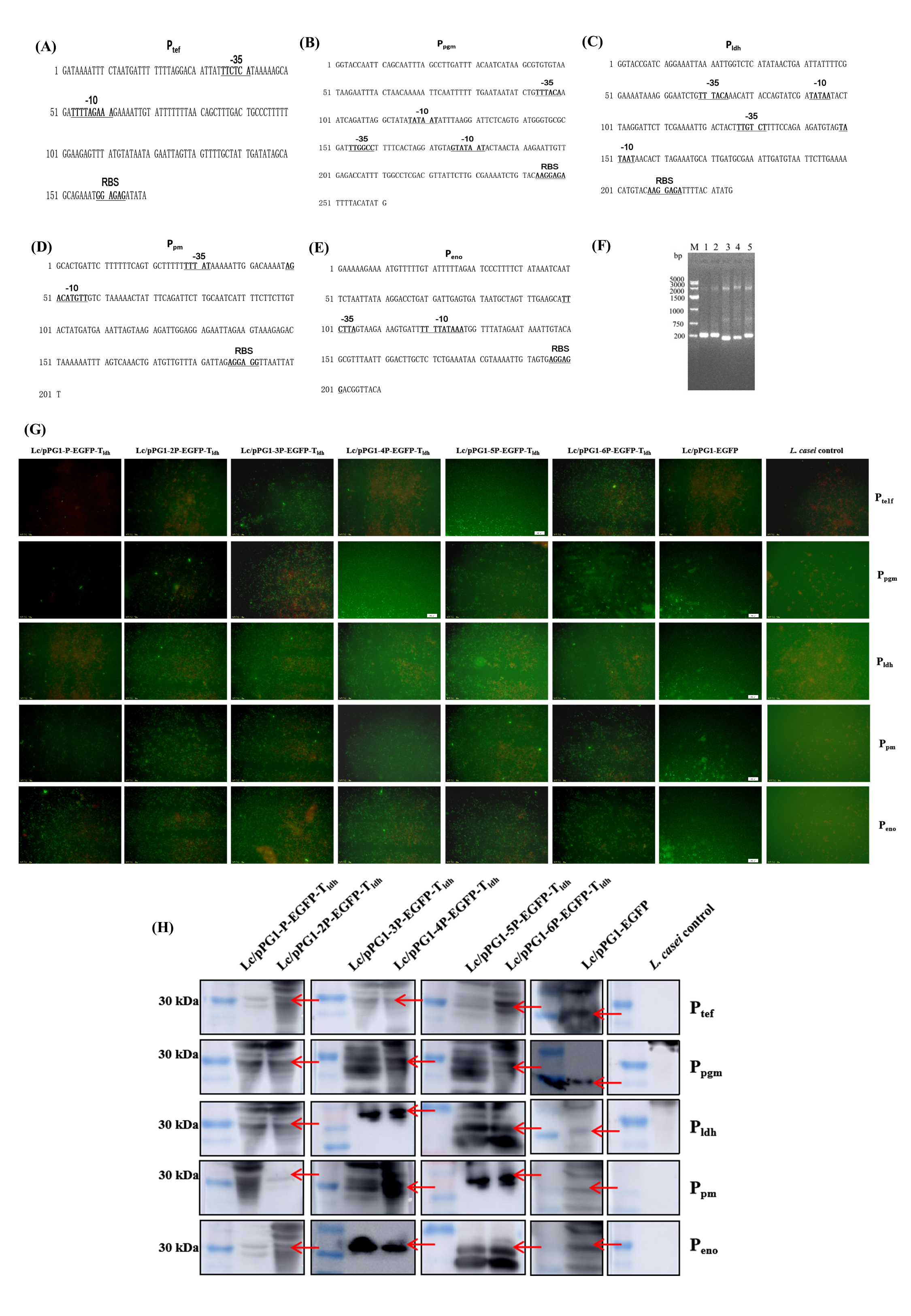
**

**Supplementary Figure 1. Validation of promoter selection and recombinant protein expression in *Lactobacillus casei*.** (A-E) Nucleotide sequences and structural features of five candidate promoters (P_tef_, P_pgm_, P_ldh_, P_pm_, and P_eno_). Critical regulatory elements (-35 boxes, -10 boxes, ribosome-binding sites) are indicated by underlining. (F) Electrophoretic verification of amplified promoter fragments. Lanes 1-5: PCR products corresponding to P_tef_ (225 bp), P_pgm_ (261 bp), P_ldh_ (169 bp), P_pm_ (201 bp) and P_eno_ (210 bp). Molecular weight: DL5000 DNA marker (5000 bp). (G) Surface localization of EGFP fusion protein confirmed by immunofluorescence microscopy (1000×). Fixed cells were probed with anti-EGFP IgG (1:100), followed by Alexa Fluor 488-conjugated secondary antibody. (H) Western blot verification of recombinant protein expression. A ~55 kDa band corresponding to the fusion construct was detected exclusively in transformed strains. All experiments were replicated three times independently.


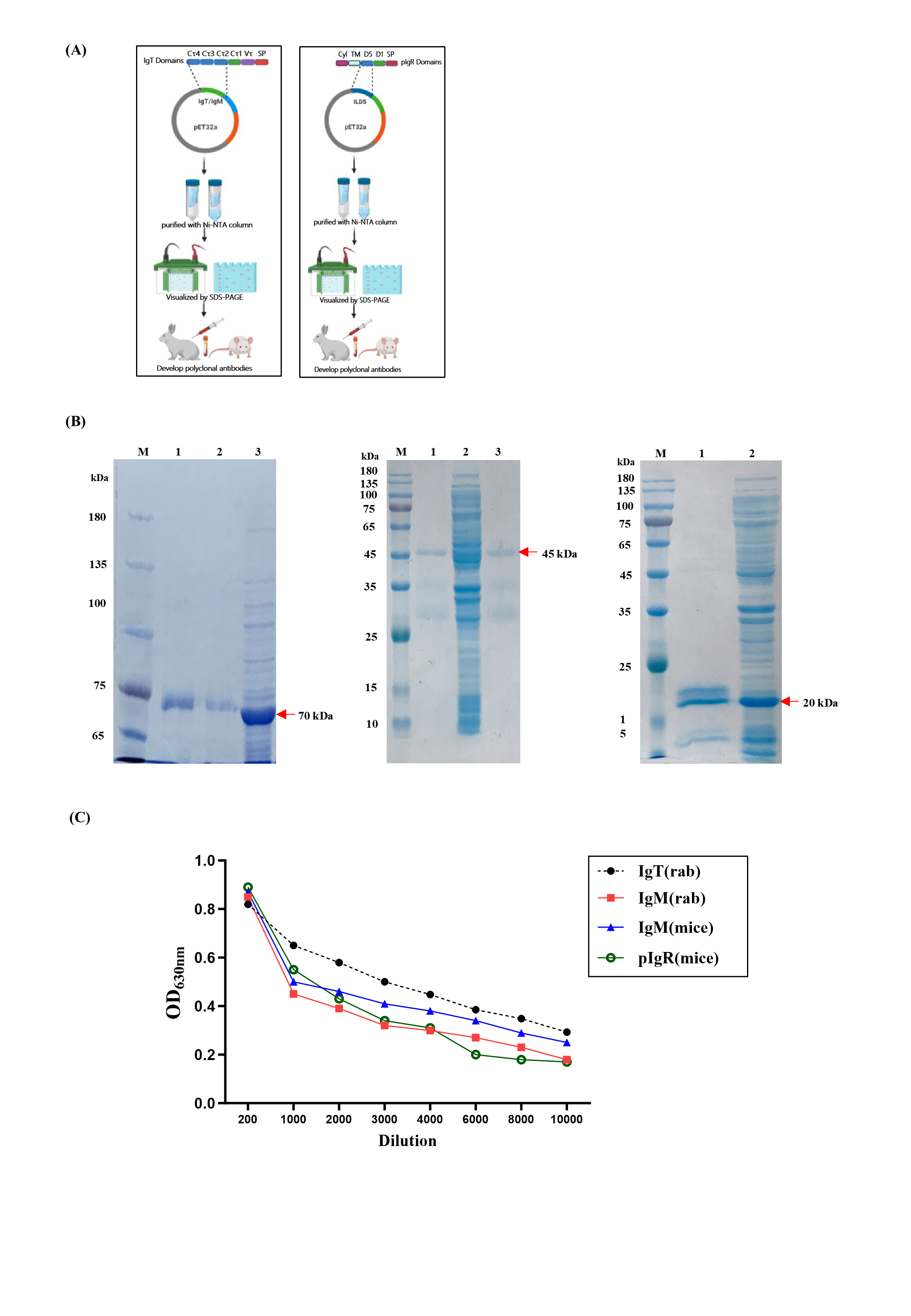


**Supplementary Figure 2. Preparation of polyclonal antibodies against IgT (CH2-4), IgM (CH2-4) and pIgR (ILD5).** (A) Prokaryotic expression of IgT, IgM, and pIgR genes and antibody preparation strategy diagram. (B) SDS-PAGE analysis of prokaryotic expression of IgT (left), IgM (middle), and pIgR (right) genes. (C) Determination of the titer of rabbit anti-snakehead IgT, IgM, and mouse anti-snakehead IgM, pIgR antibodies.

**
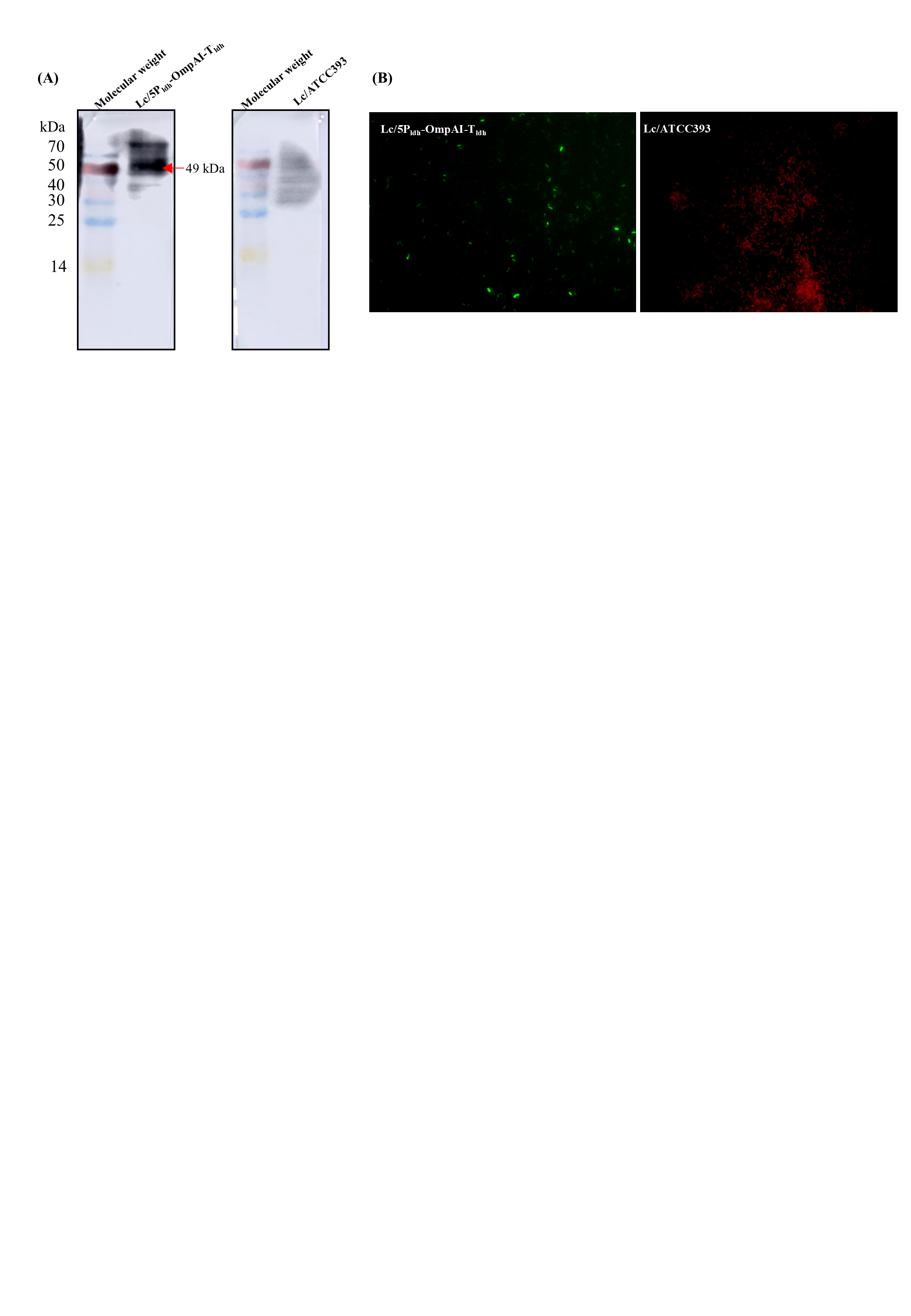
Supplementary Figure 3. Validation of surface-localized OmpAI expression in *Lactobacillus casei*.** (A) Western blot analysis of whole-cell lysates under denaturing conditions. (B) Immunofluorescence confirmation of surface expression (1000×). Left: Lc/5P_ldh_-OmpAI-T_ldh_ strain stained with anti-OmpAI IgG (1:100) and FITC conjugate. Right: Vector control strain (Lc/pPG612). Green fluorescence indicated membrane-localized OmpAI. All experiments replicated three times independently.

**
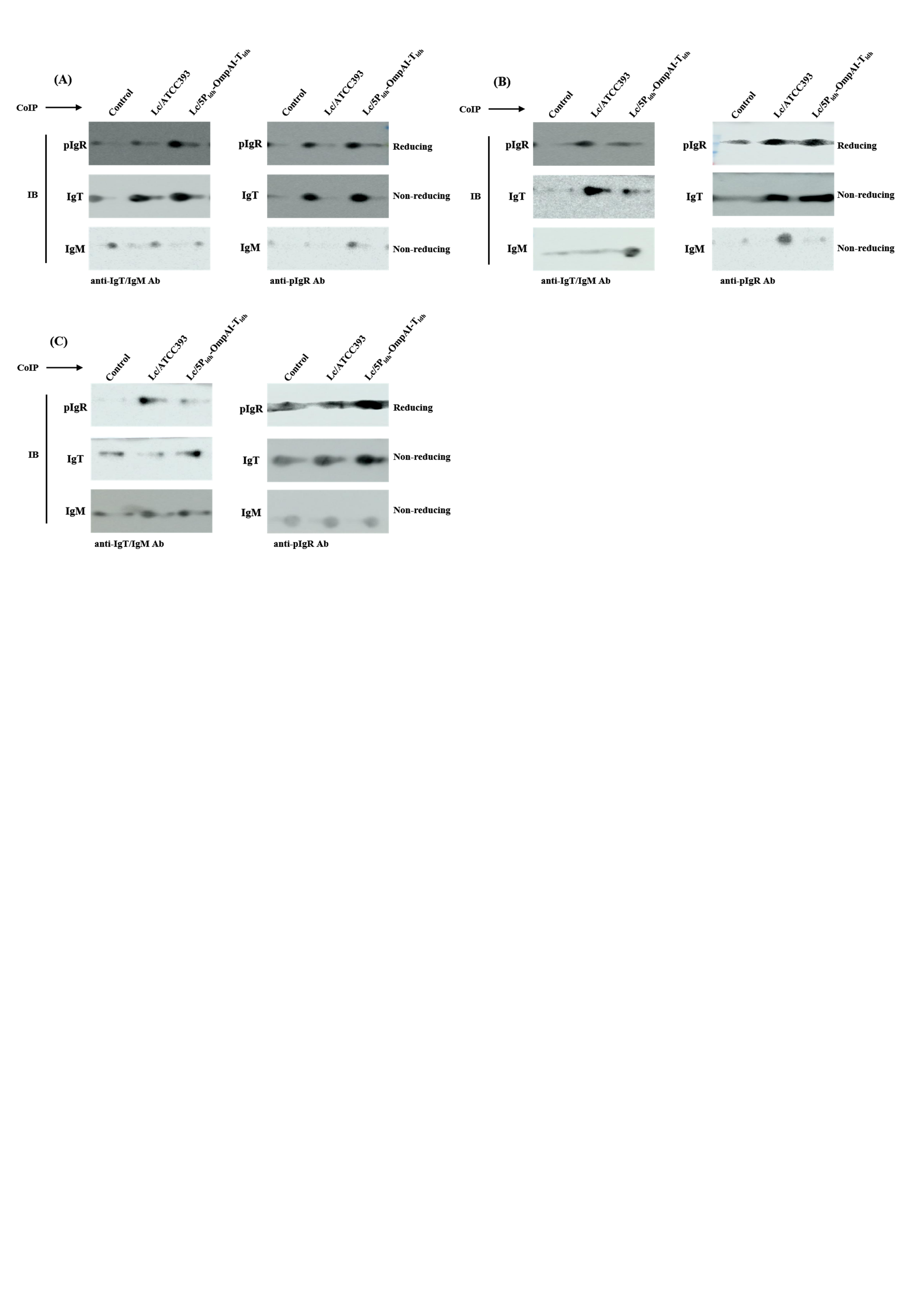
Supplementary Figure 4. Co-immunoprecipitation analysis of pIgR-mediated immunoglobulin transport in mucosal secretions of snakehead.** (A-C) Immunoblot analysis of gut (A), skin (B), and gill (C) mucus through immunoglobulin co-immunoprecipitation. Upper panels: Immunoprecipitated complexes probed with anti-pIgR polyclonal antibody (1:2000) under denaturing conditions. Lower panels: The blots probed with anti-IgT or anti-IgM polyclonal antibodies (1:2000) under non-denaturing conditions. Representative blots from three independent experiments.

**
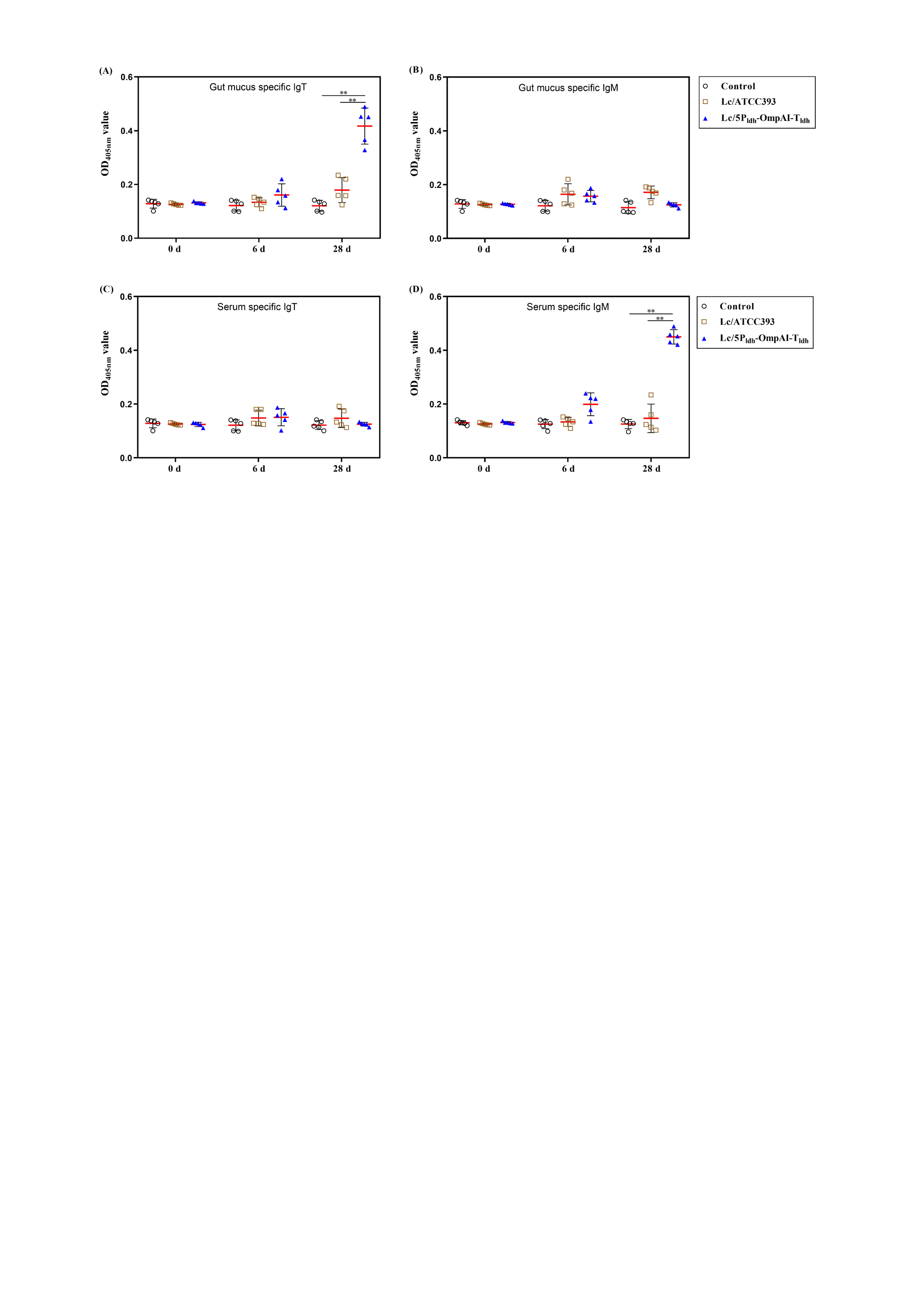
Supplementary Figure 5. Engineered *Lactobacillus casei* induced OmpAI-specific immunoglobulin responses in mucosal and systemic compartments.** (A, B) Antigen-specific antibody titers in gut mucus determined by ELISA. Serial dilutions were probed with HRP-conjugated anti-IgT (A) or anti-IgM (B) antibodies (1:5000). (C, D) Serum antibody analysis using anti-IgT (C) or anti-IgM (D) detection. Data represent mean optical density±SEM (n=5). All experiments replicated twice independently.
